# Cytotoxic Phenylpropanoid Derivatives and Alkaloids from the Flowers of *Pancratium maritimum* L.

**DOI:** 10.3390/plants11040476

**Published:** 2022-02-09

**Authors:** Diaa T. A. Youssef, Lamiaa A. Shaala, Ahmed E. Altyar

**Affiliations:** 1 Department of Natural Products, Faculty of Pharmacy, King Abdulaziz University, P.O. Box 80260, Jeddah 21589, Saudi Arabia; 2 Natural Products Unit, King Fahd Medical Research Center, King Abdulaziz University, P.O. Box 80260, Jeddah 21589, Saudi Arabia; 3 Department of Medical Laboratory Sciences, Faculty of Applied Medical Sciences, King Abdulaziz University, P.O. Box 80260, Jeddah 21589, Saudi Arabia; 4 Department of Pharmacy Practice, Faculty of Pharmacy, King Abdulaziz University, P.O. Box 80260, Jeddah 21589, Saudi Arabia; aealtyar@kau.edu.sa

**Keywords:** Amaryllidaceous plant, *Pancratium maritimum* L., flowers, glycosides, phenylpropanoid derivatives, alkaloids, cancer and normal cell lines, antiproliferation and growth inhibition

## Abstract

Regarding our growing interest in identifying biologically active leads from Amaryllidaceous plants, the flowers of *Pancratium maritimum* L. (Amaryllidaceae) were investigated. Purification of the cytotoxic fractions of the alcoholic extract of the flowers gave a new glycoside, 3-[4-(*β*-D-glucopyranosyloxy)phenyl]-2-(*Z*)-propenoic acid methyl ester (**1**), together with the previously reported compounds 3-methoxy-4-(*β*-D-glucopyranosyloxy)benzoic acid methyl ester (**2**), 3-(4-methoxyphenyl)propan-1-ol-1-*O-β*-D-glucopyranoside (**3**), (*E*)-3-(4-hydroxyphenyl)acrylic acid methyl ester (**4**), caffeic acid (**5**), dihydrocaffeic acid methyl ester (**6**), and pancratistatin (**7**). Interestingly, compounds **1** and **2** are phenolic-*O*-glycosides, while the glucose moiety in **3** is attached to the propanol side chain. This is the first report about the existence of **1**–**6** in the genus *Pancratium*. Further, glycosides **1**–**3** from the Amaryllidaceae family are reported on here for the first time. The structures of **1**–**7** were determined by analyses of their 1D (^1^H and ^13^C) and 2D (COSY, HMQC, HMBC) NMR spectra, and by high-resolution mass spectral measurements. Pancratistatin displayed potent and selective growth inhibitory effects against MDA-MB-231, HeLa, and HCT 116 cells with an IC_50_ value down to 0.058 µM, while it possessed lower selectivity towards the normal human dermal fibroblasts with IC_50_ of 6.6 µM.

## 1. Introduction

The Amaryllidaceae family consist of about 85 genera and 1100 species; they are well known for their ornamental values and biologically-active alkaloids [1]. Plants of the genus *Pancratium* are bulbous monocotyledons, which grow on sand banks and sandy coastal environments [2]. Although *Pancratium* includes about 15 species, distributed throughout the Mediterranean, Africa, and Asia, only three species of this genus, *P. maximum, P. sickenbergeri,* and *P. tortuosum,* are indigenous to Saudi Arabia [3]. In Egypt, there are four species belonging to the genus *Pancratium*, namely *P. arabicum*, *P. maritimum* L., *P. sickenbergeri,* and *P. tortuosum* [4]. The extracts of the bulbs and flowers of *P. maritimum* possess analgesic, antifungal, and anticancer activities. Furthermore, these extracts possess purgative, hypotensive, emetic, and anti-inflammatory effects [5].

Cancer is a worldwide health concern and accounts for 8 million deaths worldwide each year, with almost 600,000 deaths in the United States only [6]. Plant-derived alkaloids, such as vinblastine, vincristine, and paclitaxel, are valuable and important antitumor drugs [7].

Chemists and pharmacologists worldwide have substantially considered members of the Amaryllidaceae family, due to their complex and diverse alkaloids and biological and pharmacological properties. Amaryllidaceous alkaloids display antimalarial [8,9,10], analgesic [11], acetylcholinesterase [12], and antifungal [13] activities. After the discovery of pancratistatin [14], with its narciclasine-backbone and its significant antitumor effects [15], representative alkaloids of this class have been evaluated as potential cytotoxic agents [16]. Later on, the anticancer potential of the semisynthetic derivatives of narciclasine [17], lycorine [18], and crinine [19] were investigated. 

On the contrary, the non-alkaloidal constituents of Amaryllidaceae plants have been overlooked by researchers. However, since the identification of chromones, chromones, acetophenones, and their glycosides from *Pancratium biflorum* [20,21,22], several studies were carried out to explore the non-alkaloidal constituents of the genus *Pancratium* [23,24,25], resulting in identification of different chromones and acetophenones and their glycosides, flavonoids, chalcones, and others [20,21,22,23,24,25]. 

As a continuation of our work in exploring the secondary metabolites of Amaryllidaceous plants [26,27,28,29,30], the cytotoxic fractions of the alcoholic extracts of the fresh flowers of *P. maritimum* L. (Sea Daffodil) were investigated. One new glycoside (**1**), together with the previously reported compounds **2**–**7**, were purified from the active fractions of the flower extracts. In this work, we describe the purification, structural assignments, and the cytotoxic effects of the compounds.

## 2. Results and Discussion

### 2.1. Structure of Compound ***1***

**Figure 1:**
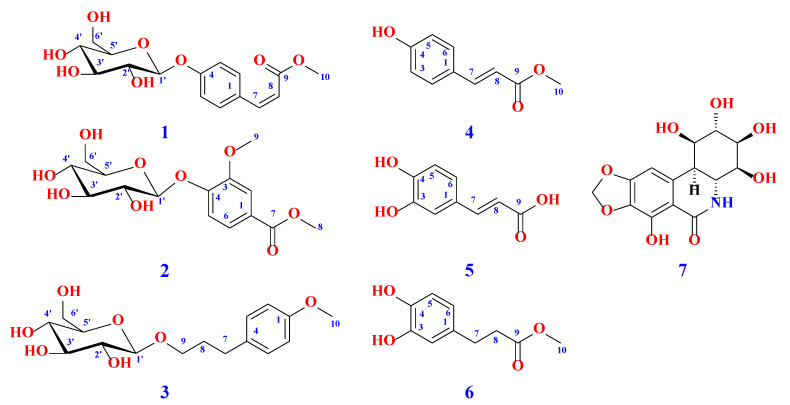
Structures of compounds **1**–**7**.

Compound **1** (Figure 1) possesses the molecular formula C_16_H_20_O_8_, as shown by a pseudomolecular ion peak at *m*/*z* 363.1055 [M + Na]^+^ in the (+)-HRESIMS (Figure S1), suggesting seven degrees of unsaturation. The structure of **1** was assigned from interpretation of its 1D (Figures S2–S5) and 2D NMR spectra (Figures S6–S8). The ^1^H and ^13^C NMR data of **1** (Table 1) showed signals for 1,4-disubstituted benzene moiety linked to Z-propenoic acid methyl ester and *O-β*-glucopyranoside units. The ^1^H and ^13^C NMR signals at δ_H/C_ 130.4 (qC, C-1), 7.68 (d, *J* = 8.7 Hz)/133.0 (2 × CH, H-2,6/C-2,6), 7.08 (d, *J* = 8.7 Hz)/117.1 (2 × CH, H-3,5/C-3,5), 159.9 (qC, C-4) supported the assignment of the 1,4-substituted benzene moiety. 

The propenoic acid methyl ester moiety was assigned from the ^1^H and ^13^C NMR resonances at δ_H/C_ 6.93 (d, *J* = 12.7 Hz)/144.3 (CH, H-7/C-7), 5.87 (d, *J* = 12.7 Hz)/117.6 (H-8/C-8), 168.6 (qC, C-9) and 3.72 (s)/51.8 (CH_3_, H_3_-10/C-10). The *Z* configuration at Δ^7,8^ was assigned from the coupling constants of 12.7 Hz between the olefinic protons H-7 and H-8. In addition, HMBC from H-7 and H-8 to C-9 and from H_3_-10 to C-9 supported the assignment.

The ^1^H/^13^C resonances at δ_H/C_ 4.98 (d, *J* = 7.2 Hz)/102.0 (CH, H-1′/C-1′), 3.48 (m)/74.9 (CH, H-2′/C-2′), 3.49 (m)/78.0 (CH, H-3′/C-3′), 3.41 (m)/71.4 (CH, H-4′/C-4′), 3.49 (m)/78.3 (CH, H-5′/C-5′) and 3.91 (t, *J* = 11.9 Hz), 3.72 (m)/62.5 (CH_2_, H_2_-6′/C-6′) proposed the presence of a glucopyranoside unit. The continuous spin–spin COSY couplings from the anomeric proton H-1′ (5.04 ppm) to H_2_-6′ (3.91 and 3.71 ppm) supported the assignment of the glucopyranoside moiety. The coupling constant of the anomeric proton (H-1′, d, *J* = 7.2 Hz) supported the *β* configuration of the glucopyranoside unit (Table 1).

The attachment of the *Z*-propenoic acid methyl ester to the benzene ring at C-1 was supported by HMBC correlations from H-7 and H-8 to C-1, and from H-2,6 to C-7. Similarly, the placement of the *β-D*-glucopyranoside moiety at C-4 of the benzene moiety was supported by HMBC cross-peaks from H-3,5 to C-1′ and from H-1′ to C-4. Thus, **1** was assigned as 4-(*β*-D-glucopyranosyloxy)phenyl]-2-(*Z*)-propenoic acid methyl ester, and it is reported here as a new natural product.

### 2.2. Structure of Compound ***2***

**Figure 2:**
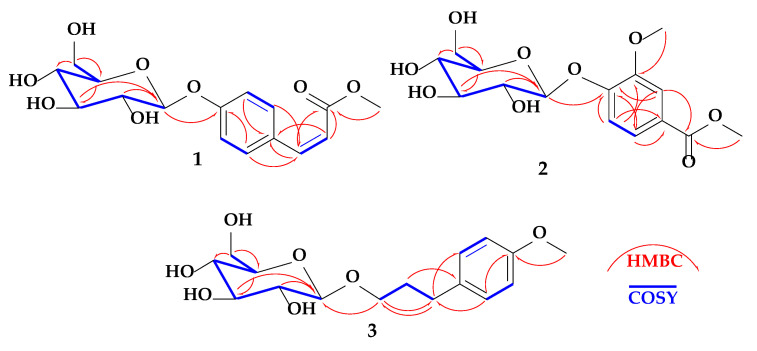
Key COSY and HMBC correlations of **1**–**3**.

Compound **2** (Figure 1) showed the molecular formula C_15_H_20_O_9_ as obtained from the pseudomolecular ion peak at *m/z* 367.1003 [M + Na]^+^ in the (+)-HRESIMS (Figure S9), suggesting six degrees of unsaturation. The structure of **2** was assigned from interpretation of its 1D (Figures S10–S13) and 2D NMR spectra (Figures S14–S16). The ^1^H and ^13^C NMR data of **2** (Table 2) showed resonances for 3,4-disubstituted benzoic acid methyl ester. The ^1^H and ^13^C NMR resonances at δ_H/C_ 125.5 (qC, C-1), 7.62 (d, *J* = 1.8 Hz)/114.1 (CH, H-2/C-2), 150.5 (qC, C-3), 152.2 (qC, C-4), 7.23 (d, *J* = 8.5 Hz)/116.5 (CH, H-5/C-5), 7.65 (dd, *J* = 8.5 and 1.8 Hz)/ 124.6 (CH, H-6/C-6), 168.3 (qC, C-7) and 3.89 (s)/52.6 (CH_3_, H_3_-8/C-8) proposed the existence of 3,4-disubstituted benzoic acid methyl ester. The COSY correlation between H-6 and H-2 and between H-6 and H-5 supported the existence of an ABX system and the assignment of these protons (Figure 2). In addition, the protonated carbons were assigned from HSQC correlations (Table 2). 

The substituents at C-3 and C-4 were assigned as a methoxyl (δ_H/C_ 3.92 (s)/56.2) group and an *O-β*-glucopyranoside unit, respectively. The presence of *O-β*-glucopyranoside moiety was established from the ^1^H and ^13^C NMR resonances at δ_H/C_ 5.04 (d, *J* = 7.7 Hz)/102.0 (CH, H-1′/C-1′), 3.55 (dd, *J* = 9.1 and 7.7 Hz)/74.8 (CH, H-2′/C-2′), 3.48 (m)/77.9 (CH, H-3′/C-3′), 3.42 (t, *J* = 9.1 Hz)/71.3 (CH, H-4′/C-4′), 3.50 (t, *J* = 9.1 Hz)/78.4 (CH, H-5′/C-5′) and 3.82 (m), 3.71 (dd, *J* = 11.0 and 4.5 Hz)/62.5 (CH_2_, H_2_-6′/C-6′). The continuous spin-spin COSY couplings from the anomeric proton H-1′ (5.04 ppm) to H_2_-6′ (3.91 and 3.71 ppm) supported the assignment of the glucopyranoside moiety. The coupling constant of the anomeric proton (H-1′, d, *J* = 7.7) supported the *β* configuration of the glucopyranoside unit (Table 2 and Figure 2).

The placement of the OCH_3_ group at C-3 was supported by HMBC correlations from H-5 to C-3 and from H_3_-9 to C-3, while the HMBC from H-1′ to C-4 and from H-5 to C-1′ secured the placement of the glucopyranoside moiety at C-4. The ^1^H and ^13^C NMR data of **2** are similar to those of 3-methoxy-4-(*β*-D-glucopyranosyloxy)benzoic acid methyl ester [31], which was previously reported from the Solanaceous plant *Lycium schweinfurthii* (family: Solanaceae) [31]. Thus, compound **2** was assigned as 3-methoxy-4-(*β*-D-glucopyranosyloxy)benzoic acid methyl ester. This is the first report about the existence of compound **2** in the Amaryllidaceae family.

### 2.3. Structure of Compound ***3***

Compound **3** (Figure 1) displayed molecular formula C_16_H_24_O_7_ as supported by the pseudomolecular ion peak at *m/z* 351.1419 [M + Na]^+^ in the (+)-HRESIMS (Figure S17), requiring five degrees of unsaturation. The structure of **3** was assigned from interpretation of its 1D (Figures S18–S21) and 2D NMR spectra (Figures S22–S24). The ^1^H and ^13^C NMR spectra of **3** displayed signals for 3-(4-methoxyphenyl)propan-1-ol linked to a *β-O*-glucopyranoside unit at the OH of the propanol side chain. The ^1^H and ^13^C NMR resonances at δ_H/C_ 159.4 (qC, C-1), 6.83 (d, *J* = 8.5 Hz)/114.8 (CH, H-2,6/C-2,6), 7.13 (d, *J* = 8.5 Hz)/130.5 (CH, H-3,5/C-3,5), 135.4 (qC, C-4), 2.67 (t, *J* = 7.6 Hz)/32.3 (CH_2_, C-7), 1.90 (m)/32.9 (CH_2_, C-8), 3.92 (dt, *J* = 9.6, 6.5) 3.54 (dt, *J* = 9.6, 6.5 Hz)/70.1 (CH_2_, C-9), and 3.77 (s)/55.7 (CH_3_, H_3_-10/C-10) were assigned as 3-(4-methoxyphenyl)propan-1-ol moiety (Table 3). 

The *β*-glucopyranoside moiety was assigned from ^1^H and ^13^C NMR signals at 4.25 (d, *J* = 7.8 Hz)/104.5 (CH, H-1′/C-1′), 3.21 (dd, *J* = 9.0 and 7.8 Hz)/75.2 (CH, H-2′/C-2′), 3.37 (t, *J* = 9.0 Hz)/78.2 (CH, H-3′/C-3′), 3.31 (t, *J* = 9.0 Hz)/71.7 (CH, H-4′/C-4′), 3.27 (m)/77.9 (CH, H-5′/C-5′) and 3.88 (dd, *J* = 11.9 and 2.2 Hz), 3.69 (dd, *J* = 11.9 and 6.1 Hz)/62.8 (CH_2_, H_2_-6′/C-6′) (Table 3). The chemical shift of the anomeric proton (H-1′) at δ_H_ 4.25 and its coupling constant of 7.8 Hz supported the *β* configuration of the glucopyranoside moiety.

The COSY experiment displayed three coupling systems, including the coupling between H-2,6 and H-3,5 in the aromatic moiety, the vicinal couplings in the aliphatic side chain from H_2_-7 to H_2_-8 and from H_2_-8 to H_2_-9, and the contiguous coupling system within the glucopyranoside moiety from H-1′ to H_2_-6′ (Figure 2). The placement of the OCH_3_ group at C-1 was assigned from HMBC of H_3_-10 to C-1 (Table 3 and Figure 2). Similarly, the attachment of the glucose moiety at the terminal OH of the propanol moiety was assigned from HMBC correlations from H_2_-9 to C-1′ and from H-1′ to C-9. The NMR data of **3** are similar to those of 3-(4′-methoxyphenyl)-propanol 1-*O*-*β*-glucopyranoside, which was previously isolated from the plant *Mediasia macrophylla* (family: Apiaceae) [32]. Thus, **3** was assigned 3-(4-methoxyphenyl)propan-1-ol-1-*O-β*-D-glucopyranoside. This is the first report about the occurrence of compound **3** in the Amaryllidaceae family.

### 2.4. Structure of Compound ***4***

Compound **4** (Figure 1) displayed molecular C_10_H_10_O_3_ as obtained from (+)-HRESIMS (Figure S25). Its ^1^H NMR spectrum (Figure S26) revealed resonances for 1,4-disubstituted benzene ring (Table 4) at δ_H_ 7.48 (2H, d, *J* = 8.5 Hz, H-2,6) and 6.82 (2H, d, *J* = 8.5 Hz, H-3,5). In addition, signals at δ_H_ 7.67 (1H, d, *J* = 15.9 Hz, H-7), 6.37 (1H, d, *J* = 15.9 Hz, H-8), and 3.73 (3H, s, H_3_-10) suggested the presence of acrylic acid methyl ester. Furthermore, the ^13^C NMR spectrum of **4** (Figure S27) displayed signals for six methines at δ_C_ 131.2 (C-2,6), 116.9 (C-3,C-5), 146.5 (C-7), and 115.9 (C-8), one methoxyl at δ_C_ 52.9 (C-10), and three quaternary carbons at δ_C_ 127.4 (C-1), 161.2 (C-4) and 169.0 (C-9). These ^1^H and ^13^C NMR data are similar to those of (*E*)-3-(4-hydroxyphenyl)acrylic acid methyl ester [33], which was previously isolated from the mangrove-derived actinobacterium *Saccharomonospora oceani* VJDS-3 [33]. Thus, **4** was assigned as (*E*)-3-(4-hydroxyphenyl)acrylic acid methyl ester. This is the first report about the existence of this compound in the genus *Pancratium*.

### 2.5. Structure of Compound ***5***

Compound **5** (Figure 1) displayed molecular formula C_9_H_8_O_4_ as supported from (+)-HRESIMS (Figure S28). Its ^1^H NMR spectrum (Figure S29) revealed resonances for five protons in the olefinic/aromatic region (Table 4). The ^1^H signals are counted for an ABX system (H-2, H-5 and H-6) at δ_H_ 7.05 (d, *J* = 1.5 Hz, H-2), 6.79 (d, *J* = 8.0 Hz, H-5) and 6.94 (dd, *J* = 8.0 and 1.5 Hz, H-6) and signals for an *E*-configured double bond (H-7 and H-8) at δ_H_ 7.54 (d, J = 15.8 Hz, H-7) and 6.24 (d, *J* = 15.8 Hz, H-8). Furthermore, the ^13^C NMR spectrum of **5** (Figure S30) showed resonances for nine carbons including five methines at δ_C_ 115.1, 116.5, 122.8, 146.9, and 116.0 are assigned for C-2, C-5, C-6, C-7, and C-8, respectively, and four quaternary carbons at 127.9 (C-1), 146.8 (C-3), 149.4 (C-4), and 171.1 (C-9) (Table 4). These ^1^H and ^13^C NMR data are similar to those reported for caffeic acid (3,4-dihydroxycinnamic acid) [34], which was isolated before from Hippeastrum vittatum (family: Amaryllidaceae) [34]. Thus, **5** was assigned as caffeic acid (3,4-dihydroxycinnamic acid). This is the first occurrence of this compound in the genus *Pancratium*.

### 2.6. Structure of Compound ***6***

Compound **6** (Figure 1) displayed molecular C_10_H_12_O_4_ as supported from (+)-HRESIMS (Figure S31). Its ^1^H NMR spectrum (Figure S32) revealed resonances for an ABX system (H-2, H-5 and H-6) at δ_H_ 6.63 (d, *J* = 1.9 Hz, H-2), 6.68 (d, *J* = 8.0 Hz, H-5) and 6.51 (dd, *J* = 8.0 and 1.9 Hz, H-6) and signals for an ethylene moiety (H_2_-7 and H_2_-8) at δ_H_ 2.77 (t, *J* = 7.6 Hz, H_2_-7) and 2.57 (t, *J* = 7.6 Hz, H_2_-8) and one methoxyl at δ_H_ 3.65 (s, 3H, H_3_-10). Furthermore, the ^13^C NMR spectrum of **6** (Figure S33) showed resonances for nine carbons including three methines at δ_C_ 116.5, 116.4, 120.5 assigned for C-2, C-5, C-6, respectively, two methylenes at 31.1 (C-7) and 37.1 (C-8), one methoxyl at δ_C_ 52.1 (C-10), and four quaternary carbons at 133.6 (C-1), 146.3 (C-3), 144.7 (C-4), and 175.4 (C-10) (Table 4). These ^1^H and ^13^C NMR data are similar to those reported for dihydrocaffeic acid methyl ester, which was isolated from *Hippeastrum vittatum* (family: Amaryllidaceae) [34]. Thus, **6** was assigned as dihydrocaffeic acid methyl ester. This is the first report about the existence of this compound in the genus *Pancratium*.

### 2.7. Structure of Compound ***7***

Compound **7** (Figure 1) possesses molecular formula C_14_H_15_NO_8_ as supported from the pseudo-molecular ion peak at *m/z* 348.0689 [M + Na]^+^ in the (+)-HRESIMS (Figure S34). The ^1^H and ^13^C NMR data (Figures S35 and S36) of **7** are similar to those reported for (+)-pancratistatin [14,35,36], which was isolated previously from *Pancratium littorale* (family: Amaryllidaceae). Accordingly, **7** was assigned as pancratistatin.

### 2.8. Antiproliferation Activities of the Compounds

Compounds **1**–**7** were evaluated for their antiproliferative and growth inhibition activities against MDA-MB-231, HeLa, and HCT 116 cell lines (Table 5). Pancratistatin displayed a potent growth inhibitory activity towards these cell lines with IC_50_ values 0.14, 0.058, and 0.10 μM, respectively. On the other hand, compounds **1**–**3** and **5** were inactive at the level of 10 μM against these cells. Due to the high potency of compound **7**, a concentration of 10 µM was set as a cutoff value in this assay.

**Figure 3:**
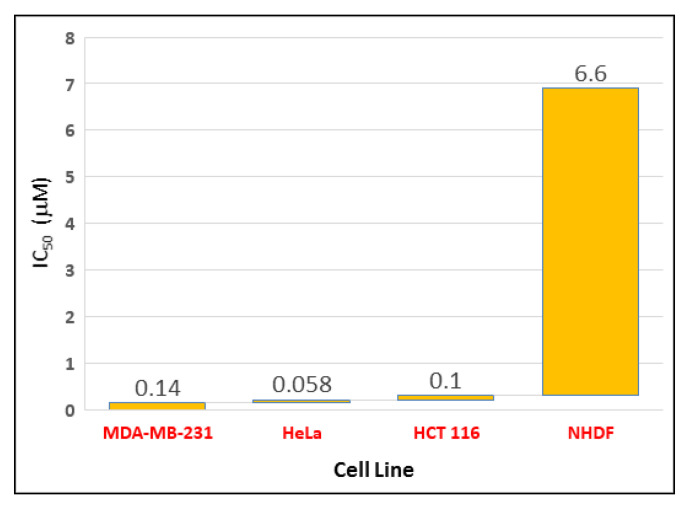
Comparison of the growth inhibitory effects of pancratistatin (**7**) against cancer cell lines versus NHDF.

To evaluate the selectivity of pancratistatin against cancer cell lines, it was evaluated against the normal human dermal fibroblasts (NHDF). Pancratistatin displayed less selectivity towards NHDF with IC_50_ of 6.6 µM, suggesting a higher selectivity (66-fold) towards the tested cancer cell lines (Table 5 and Figure 3).

It is well known that pancratistatin possess a wide-range selectivity towards various cancer cell lines [14,37,38] with IC_50_ ranging from 0.043 to 0.098 µM [37,38]. Furthermore, pancratistatin induce apoptosis through targeting mitochondria in cancer cells [39,40]. Moreover, it causes flipping of phosphatidyl-serine, activation of caspase-3, generation of reactive oxygen species (ROS), and loss of the mitochondrial membrane leading to apoptosis [41].

However, it is worth mentioning that this is the first report, to the best of our knowledge, about the evaluation of pancratistatin against MDA-MB-231, HeLa, HCT 116, and NHDF cell lines.

## 3. Materials and Methods

### 3.1. General Experimental Procedures 

Optical rotations of the compounds were measured on a JASCO DIP-370 digital polarimeter at 25 °C at the sodium D-line (589 nm). The IR spectra were recorded on a Shimadzu Infrared-400 spectrophotometer (Shimadzu, Kyoto, Japan). The 1D and 2D NMR spectra (chemical shifts in ppm, coupling constants in Hz) were recorded on Bruker Avance DRX 600 MHz (600 MHz for ^1^H and 150 MHz for ^13^C NMR) (Bruker, Rheinstetten, Germany) and Bruker Ascend 850 MHz (850 MHz for ^1^H and 213 MHz for ^13^C NMR) (Bruker BioSpin, Billerica, MA, USA) spectrometers. Positive ion HRESIMS data were obtained with a Micromass Q-Tof equipped with leucine enkephalin lock spray, using *m*/*z* 556.2771 [M + H]^+^ as a reference mass. Sephadex LH-20 (0.25–0.1 mm, Pharmacia) was used for column chromatography. Precoated silica gel 60 F-254 plates (Merck) were used for TLC. HPLC purifications were performed on Shim-Pack, PREP-ODS (H) (20 × 250 mm), Cosmosil ARII C18 (20 × 250 mm), and *Atlantis* Prep OBD T3 Column (10 × 250 mm, 5 µm).

### 3.2. Botanical Materials

**Figure 4:**
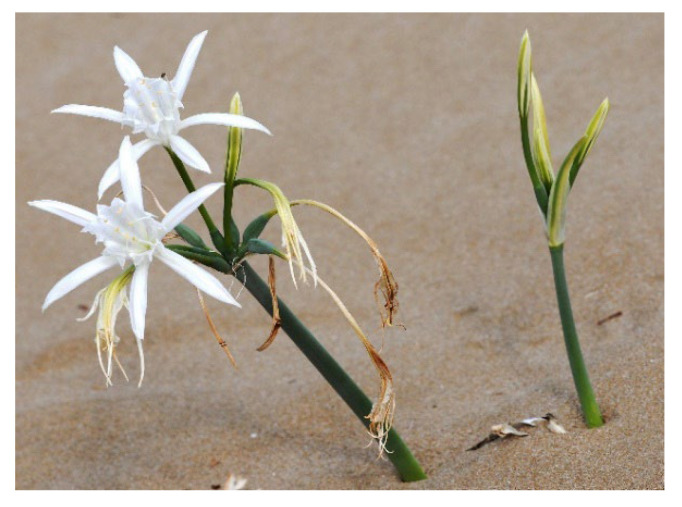
Photograph of the flowers of *Pancratium maritimum* L.

The fresh entire flowers of *P. maritimum* L. with most of their stalks (Figure 4) were collected from the coastal sandy beaches of the Egyptian Governorate Maṭrūḥ, Egypt. The plant materials were kindly identified at the Department of Botany, Faculty of Science, Suez Canal University, Ismailia, Egypt. A voucher specimen was kept at the Department of Pharmacognosy, Faculty of Pharmacy, Suez Canal University under code number DYPM-1.

### 3.3. Purification of Compounds ***1**–**7***

**Scheme 1:**
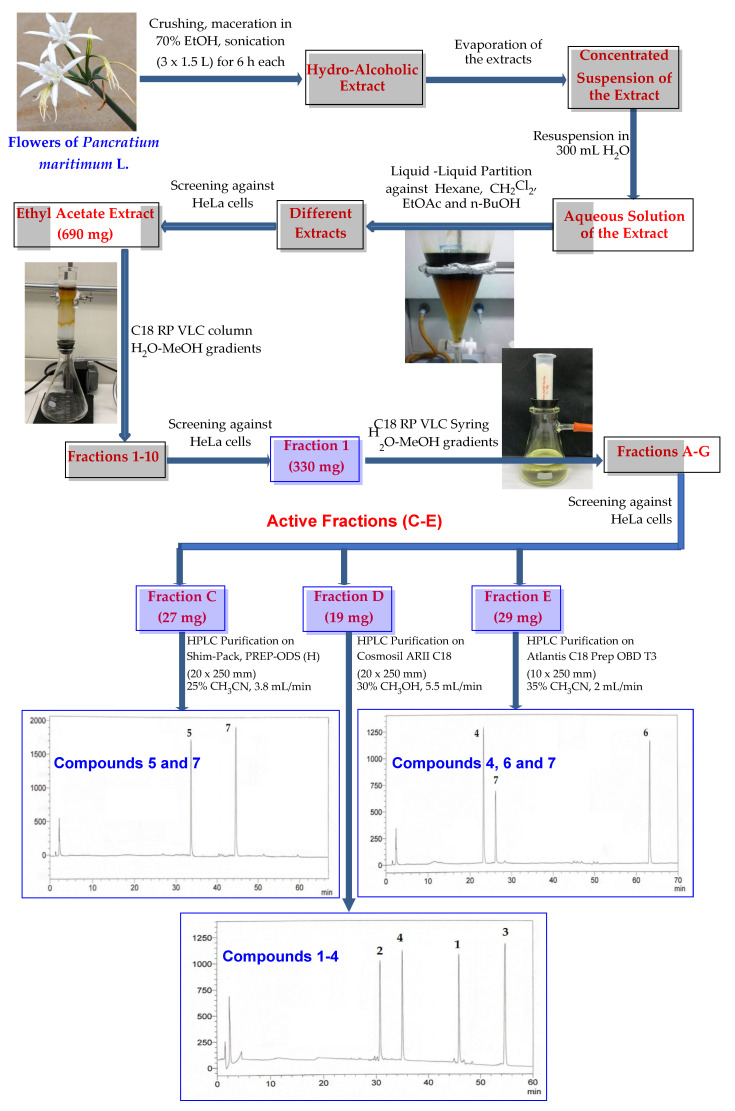
Fractionation of the extract the flowers of *P. maritimum* L. and purification of **1**–**7**.

The fresh flowers with their stalks (1.8 kg) were crushed into small pieces and soaked directly in 70% ethanol (1.5 L) (Scheme 1). After receiving the materials in the laboratory, the mixture was sonicated for an additional 6 h at room temperature and the alcoholic solution was filtered. This process was repeated thrice and the combined hydro-alcoholic extracts were evaporated under reduced pressure. The resulting suspension was dissolved in 300 mL H_2_O and successively extracted with n-hexane (3 × 200 mL), CH_2_Cl_2_ (3 × 200 mL), and ethyl acetate (3 × 200 mL). Finally, 500 mL of H_2_O were added to the remaining mother aqueous layer before gentle shaking with n-BuOH (3 × 250 mL), since n-butanol has some solubility in water. The inactive fractions (n-hexane, CH_2_Cl_2_, and n-BuOH) were kept for any future investigations. The cytotoxic ethyl acetate residue (690 mg) was chromatographed on RP C18 silica column (30 × 4 cm) using H_2_O-MeOH gradients to give 10 fractions (Fractions 1-10). The cytotoxic fraction (Fraction 1, 330 mg), which was eluted with 80% H_2_O (IC_50_ = 4.4 μg/mL against HeLa cells), was repartitioned on Sep-Pak ODS Cartridge (10 g, Waters) using H_2_O-MeOH gradients to afford seven fractions (Fractions A-G) (Scheme 1). The cytotoxic fraction C (27 mg) (IC_50_ = 1.95 μg/mL against HeLa cells), which was eluted with 20% MeOH, was purified on HPLC column (Shim-Pack, PREP-ODS (H), 20 × 250 mm) using 25% CH_3_CN at 3.8 mL/min to give compounds **5** (*t*_R_ = 34.8 min, 3.1 mg) and **7** (*t*_R_ = 44.6 min, 2.3 mg) (Figure S37). Similarly, the cytotoxic fraction D (19 mg) (IC_50_ = 2.12 μg/mL against HeLa cells), which was eluted with 30% MeOH, was purified on RP C18 HPLC column (Cosmosil ARII, 20 × 250 mm) using 30% MeOH at 5.5 mL/min to give compounds **4** (*t_R_* = 23.3 min, 7.4 mg) and **7** (*t_R_* = 26.1 min, 2.4 mg) and **6** (*t_R_* = 63.3 min, 1.9 mg) (Figure S38). Finally, the less cytotoxic fraction E (29 mg) (IC_50_ = 13.2 μg/mL against HeLa cells), which was eluted with 40% MeOH, was purified on RP C18 HPLC column (*Atlantis* Prep OBD T3 Column, 10 × 250 mm, 5 µm) using 35% CH_3_CN at 2 mL/min to give compounds **2** (*t_R_* = 31.3 min, 3.3 mg), **4** (*t_R_* = 35.1 min, 3.7 mg), **1** (*t_R_* = 46.0 min, 3.8 mg), **3** (*t_R_* = 55.1 min, 4.1 mg) (Figure S39 and Scheme 1). 

### 3.4. Spectroscopic Data of Compounds ***1**–**7***

#### 3.4.1. 3-[4-(*β-D*-glucopyranosyloxy)phenyl]-2-(*Z*)-propenoic Acid Methyl Ester (**1**)

Yellow powder; [α]_D_—31.5° (MeOH, *c* = 0.1); IR *ν*_max_ (film) 3345, 1665, 1600, 1035 cm^−1^; NMR data: see Table 2; (+)-HRESIMS *m*/*z* 363.1055 (calcd for C_16_H_20_O_8_Na [M + Na]^+^, 363.1050). 

#### 3.4.2. 3-Methoxy-4-(*β-D*-glucopyranosyloxy)benzoic Acid Methyl Ester (**2**)

White powder; [α]_D_—23.9° (MeOH, *c* = 0.1); IR *ν*_max_ (film) 3350, 1700, 1035 cm^−1^; NMR data: see Table 1; (+)-HRESIMS *m*/*z* 367.1003 (calcd for C_15_H_20_O_9_Na [M + Na]^+^, 367.0999). 

#### 3.4.3. 3-(4-Methoxyphenyl)propan-1-ol-1-*O-β*-D-glucopyranoside (**3**)

Yellowish powder; [α]_D_—27.5° (MeOH, *c* = 0.1); IR *ν*_max_ (film) 3455, 1647, 1595, 627 cm^−1^; NMR data: see Table 3; (+)-HRESIMS *m*/*z* 351.1419 (calcd for C_16_H_24_O_7_Na [M + Na]^+^, 351.1414). 

#### 3.4.4. (*E*)-3-(4-Hydroxyphenyl)acrylic Acid Methyl Ester (**4**)

Amorphous powder; IR *ν*_max_ (film) 2938, 1687, 1599 cm^−1^; NMR data: see Table 4; HRESIMS *m*/*z* 179.0709 (calcd for C_10_H_11_O_3_ [M + H]^+^, 179.0607). 

#### 3.4.5. Caffeic Acid (5)

Off-white solid; IR *ν*_max_ (film) 3421, 1665, 1518, 1025, 627 cm^−1^; NMR data: see Table S4; (+)-HRESIMS *m*/*z* 181.0501 (calcd for C_9_H_9_O_4_ [M + H]^+^, 181.0495). 

#### 3.4.6. Dihydrocaffeic Acid Methyl Ester (**6**)

Off-white powder; IR *ν*_max_ (film) 3385, 1647, 630 cm^−1^; NMR data: see Table 4; (+)-HRESIMS *m*/*z* 197.0815 (calcd for C_10_H_13_O_4_ [M + H]^+^, 197.0808). 

#### 3.4.7. Pancratistatin (**7**)

White powder; [α]_D_ + 48.5° (DMSO, *c* = 0.1); UV (MeOH) λmax: 280, 237 nm. IR (film) *ν*_max_: 3419, 1672, 1629, 1468, 1358, 1083, 1047 cm^−1^; NMR data: see Figures S28 and S29. (+)-HRESIMS *m*/*z* 348.0693 (calcd for C_14_H_15_NO_8_Na [M + Na]^+^, 348.0689). 

### 3.5. Antiproliferative and Growth Inhibition Effects of Compounds ***1**–**7***

#### 3.5.1. Culture of Cell Lines

HCT116 (Colorectal carcinoma, ATCC CCL-247) and HeLa (human cervical carcinoma, ATCC CCL-2) cells were cultured in RPMI 1640 medium with 10% FBS, and 1% penicillin–streptomycin, while MDA-MB-231 cells (triple-negative breast cancer, ATCC HTB-26) were cultured in DMEM medium with 1% penicillin–streptomycin and 10% FBS.

The normal human dermal fibroblasts (NHDF) cells were cultured in fibroblast basal medium complemented with penicillin–streptomycin (1% *v/v*), insulin (5 µg/mL), heat-inactivated fetal bovine serum (FBS) (2% *v/v*), and basic fibroblast growth factor (1 ng/mL).

#### 3.5.2. Evaluation of the Antiproliferative Activity

The evaluation of the antiproliferative effects compounds **1**–**7** were performed using MTT assay, as reported earlier [42,43,44]. In short, the cells were incubated at 37 °C overnight in 5% CO_2_/air. After that, the compounds were added at the top row of a 96-well microtiter plate; a descendant serial dilution (1:4) of the concentration was performed followed by incubation of the cells with the compounds for 72 h. Using the Cell Titer 96 AQueous non-radioactive cell proliferation protocol, the cell viability was estimated at 490 nm on a Molecular Devices Emax microplate reader. The IC_50_ values of the compounds (expressed in μM) were evaluated using the program SoftMax Pro. A concentration of 10 µM was set as a cutoff value in this assay. 

## 4. Conclusions

Partition of the cytotoxic fractions of the alcoholic extract of the fresh flowers of *P. maritimum* L. afforded a potent cytotoxic alkaloid, pancratistatin (**7**), along with three glycosides (**1–3**) and three phenylpropanoid derivatives (**4**–**6**). Their structures were determined by analyses of their NMR and HRESIMS spectral data. We should note that compound **1** is reported here as a new natural product, while compounds **2** and **3** are reported here for the first time from the Amaryllidaceae family. On the contrary, **4**–**6** are reported here for the first time from the genus *Pancratium.*

Pancratistatin displayed highly potent activity against the HCT116, HeLa, and MDA-MB-231 cell lines down to 0.058 µM. Furthermore, pancratistatin showed less selectivity towards the normal human dermal fibroblasts (NHDF) with a IC_50_ value of 6.6 µM compared to the average IC_50_ value of 0.099 µM against the cancerous cell lines HCT116, HeLa, and MDA-MB-231, suggesting a higher selectivity (66-fold) towards these cancerous cells. 

On the other hand, compounds **1**–**3** and **5** were inactive against these cell lines at a concentration of 10 µM. The results clearly indicate that pancratistatin is a powerful lead with potent antiproliferative and growth inhibitory activities against different cancer cell lines.

The results of the current study highlight the importance of medicinal plants as a great source of bioactive compounds. In the long-term, the results of this study and similar studies will have broader and long-term impacts, including opening new channels of cooperation with the private sector in the areas of biotechnology and drug discovery, and exploring indigenous plants for their chemical diversity and biomedical importance.

## Figures and Tables

**Table 1:** NMR data of **1** (850 MHz for ^1^H and 213 for ^13^C, CD_3_OD).

Position	δ_C_, Type	δ_H_ (Mult., *J* in Hz)	HMBC
1	130.4, C		
2	133.0, CH	7.68 (d, 8.7)	C-4, C-7
3	117.1, CH	7.08 (d, 8.7)	C-1
4	159.9, C		
5	117.1, CH	7.08 (d, 8.7)	C-1
6	133.0, CH	7.68 (d, 8.7)	C-4, C-7
7	144.3, CH	6.93 (d, 12.7)	C-2, C-6, C-9
8	117.6, CH	5.87 (d, 12.7)	C-1, C-9
9	168.6, C		
10	51.8, CH_3_	3.72 (s)	C-9
1′	102.0, CH	4.98 (d, 7.2)	C-4
2′	74.9, CH	3.48 (m)	C-1′
3′	78.0, CH	3.49 (m)	C-1′
4′	71.4, CH	3.41 (m)	C-6′
5′	78.3, CH	3.49 (m)	C-1′, C-3′
6′	62.5, CH_2_	3.91 (t, 11.9), 3.72 (m)	C-4′

**Table 2:** NMR data of **2** (850 MHz for ^1^H and 213 for ^13^C, CD_3_OD).

Position	δ_C_, Type	δ_H_ (Mult., *J* in Hz)	HMBC
1	125.5, C		
2	114.1, CH	7.62 (d, 1.8)	C-3, C-6, C-7
3	150.5, C		
4	152.2, C		
5	116.5, CH	7.23 (d, 8.5)	C-1, C-3, C-4
6	124.6, CH	7.65 (dd, 8.5, 1.8)	C-1, C-4, C-7
7	168.3, C		
8	52.6, CH_3_	3.89 (s)	C-7
9	56.7, CH_3_	3.92 (s)	C-3, C-1′
1′	102.0, CH	5.04 (d, 7.7)	C-4
2′	74.8, CH	3.55 (dd, 9.1, 7.7)	C-3′
3′	77.9, CH	3.48 (m)	C-1′
4′	71.3, CH	3.42 (t, 9.1)	C-3′, C-5′
5′	78.4, CH	3.50 (t, 9.1)	C-1′
6′	62.5, CH_2_	3.82 (m), 3.71 (dd, 11.0, 4.5)	C-5′

**Table 3:** NMR data of **3** (850 MHz for ^1^H and 213 for ^13^C, CD_3_OD).

Position	δ_C_, Type	δ_H_ (Mult., *J* in Hz)	HMBC
1	159.4, C		
2	114.8, CH	6.83 (d, 8.5)	C-1, C-4
3	130.5, CH	7.13 (d, 8.5)	C-1, C-7
4	135.4, C		
5	130.5, CH	7.13 (d, 8.5)	C-1, C-7
6	114.8, CH	6.83 (d, 8.5)	C-1
7	32.3, CH_2_	2.67 (t, 7.6)	C-4, C-8, C-9
8	32.9, CH_2_	1.90 (m)	C4, C-7, C-9
9	70.1, CH_2_	3.92 (dt, 9.6, 6.5), 3.54 (dt, 9.6, 6.5)	C-7, C-8, C-1′
10	55.7, CH_3_	3.77 (s)	C-1
1′	104.5, CH	4.25 (d, 7.8)	C-9, C-3′
2′	75.2, CH	3.21 (dd, 9.0, 7.8)	C-1′, C-3′
3′	78.2, CH	3.37 (t, 9.0)	C-1′
4′	71.7, CH	3.31 (t, 9.0)	C-3′, C-5′, C-6′
5′	77.9, CH	3.27 (m)	C-3′, C-6′
6′	62.8, CH_2_	3.88 (dd, 11.9, 2.2), 3.69 (dd, 11.9, 6.1)	

**Table 4:** NMR data of **4–6** (850 MHz for ^1^H and 213 for ^13^C, CD_3_OD).

Position	δ_C_, Type	δ_H_ (Mult., *J* in Hz)	δ_C_, Type	δ_H_ (Mult., *J* in Hz)	δ_C_, Type ^a^	δ_H_ (Mult., *J* in Hz) ^a^
1	127.4, C		127.9, C		133.6, C	
2	131.2, CH	7.48 (d, 8.5)	115.1, CH	7.05 (d, 1.5)	116.5, CH	6.63 (d, 1.9)
3	116.9, CH	6.82 (d, 8.5)	146.8, C		146.3, C	
4	161.2, C		149.4, C		144.7, C	
5	116.9, CH	6.82 (d, 8.5)	116.5, CH	6.79 (d, 8.0)	116.4, CH	6.68 (d, 8.0)
6	131.2, CH	7.48 (d, 8.5)	122.8, CH	6.94 (dd, 8.0, 1.5)	120.5, CH	6.51 (dd, 8.0, 1.9)
7	146.5, CH	7.67 (d, 15.9)	146.9, CH	7.54 (d, 15.8)	31.1, CH_2_	2.77 (t, 7.6)
8	115.9, CH	6.37 (d, 15.9)	116.0, CH	6.24 (d, 15.8)	37.1, CH_2_	2.57 (t, 7.6)
9	169.0, C		171.1, C		175.4, C	
10	52.9, CH_3_	3.73 (s)			52.1, CH_3_	3.65 (s)

^a^ Data acquired at 600 MHZ for ^1^H and 150 MHz for ^13^C.

**Table 5:** Antiproliferative and growth inhibition effects of **1**–**7**.

Compound	IC_50_ (μM) (Mean + SEM) ^a^			
	MDA-MB-231	HeLa	HCT 116	NHDF
1	≥10.0	≥10.0	≥10.0	NT
2	≥10.0	≥10.0	≥10.0	NT
3	≥10.0	≥10.0	≥10.0	NT
4	NT	NT	NT	NT
5	≥10.0	≥10.0	≥10.0	NT
6	NT	NT	NT	NT
7	0.14 ± 0.002	0.058 ± 0.001	0.10 ± 0.005	6.6 ± 0.034
5-FU ^b^	13.0 ± 0.30	12.3 ± 0.25	4.6 ± 0.23	NT

(^a^) The results are the mean of three independent experiments; (^b^) 5-Fluorouracil, a positive drug; NHDF: normal human dermal fibroblasts.

## Data Availability

Data is contained within the article or Supplementary Material.

## Supplementary Materials

The following are available online at https://www.mdpi.com/article/10.3390/plants11040476/s1, Figures S1–S39: (+)-HRESIMS, ^1^H NMR, ^13^C NMR, DEPT, COSY, HSQC, and HMBC spectra of compounds **1**–**7**, and HPLC chromatograms for purification of **1****–7**.
