# Supplementary material for: Cytotoxic Phenylpropanoid Derivatives and Alkaloids from the Flowers of Pancratium maritimum L
Source: Plants (Basel). 2022 Feb 9;11(4):476. doi: 10.3390/plants11040476 (PMC8875508; doi:10.3390/plants11040476)

Supporting Information

Figure S1. (+)-HRESIMS spectrum of compound **1** ········ 1
Figure S2. ^{1}H NMR spectrum of compound **1** ········ 2
Figure S3. Expansion of ^{1}H NMR spectrum of compound **1** ········ 2
Figure S4. ^{13}C NMR spectrum of compound **1** ········ 3
Figure S5. DEPT spectrum of compound **1** ········ 3
Figure S6. COSY spectrum of compound **1** ········ 4
Figure S7. Multiplicity-edited HSQC spectrum of compound **1** ········ 4
Figure S8. ^{1}H-^{13}C HMBC spectrum of compound **1** ········ 5
Figure S9. (+)-HRESIMS spectrum of compound **2** ········ 6
Figure S10. ^{1}H NMR spectrum of compound **2** ········ 7
Figure S11. Expansion of ^{1}H NMR spectrum of compound **2** ········ 7
Figure S12. ^{13}C NMR spectrum of compound **2** ········ 8
Figure S13. DEPT spectrum of compound **2** ········ 8
Figure S14. COSY spectrum of compound **2** ········ 9
Figure S15. Multiplicity-edited HSQC spectrum of compound **2** ········ 9
Figure S16. ^{1}H-^{13}C HMBC spectrum of compound **2** ········ 10
Figure S17. (+)-HRESIMS spectrum of compound **3** ········ 11
Figure S18. ^{1}H NMR spectrum of compound **3** ········ 12
Figure S19. Expansion of ^{1}H NMR spectrum of compound **3** ········ 12
Figure S20. ^{13}C NMR spectrum of compound **3** ········ 13
Figure S21. DEPT spectrum of compound **3** ········ 13
Figure S22. COSY spectrum of compound **3** ········ 14
Figure S23. Multiplicity-edited HSQC spectrum of compound **3** ········ 14
Figure S24. ^{1}H-^{13}C HMBC spectrum of compound **3** ········ 15
Figure S25. (+)-HRESIMS spectrum of compound **4** ········ 16
Figure S26. ^{1}H NMR spectrum of compound **4** ········ 17
Figure S27. ^{13}C NMR spectrum of compound **4** ········ 18
Figure S28. (+)-HRESIMS spectrum of compound **5** ········ 19
Figure S29. ^{1}H NMR spectrum of compound **5** ········ 20
Figure S30. ^{13}C NMR spectrum of compound **5** ········ 21
Figure S31. (+)-HRESIMS spectrum of compound **6** ········ 22
Figure S32. ^{1}H NMR spectrum of compound **6** ········ 23
Figure S33. ^{13}C NMR spectrum of compound **6** ········ 24
Figure S34. (+)-HRESIMS spectrum of compound **7** ········ 25
Figure S35. ^{1}H NMR spectrum of compound **7** ········ 26
Figure S36. ^{13}C NMR spectrum of compound **7** ········ 27
Figure S37. HPLC purification of Fraction C ········ 28
Figure S38. HPLC purification of Fraction D ········ 28
Figure S39. HPLC purification of Fraction E ········ 29

Figure S1. (+)-HRESIMS spectrum of compound **1**.

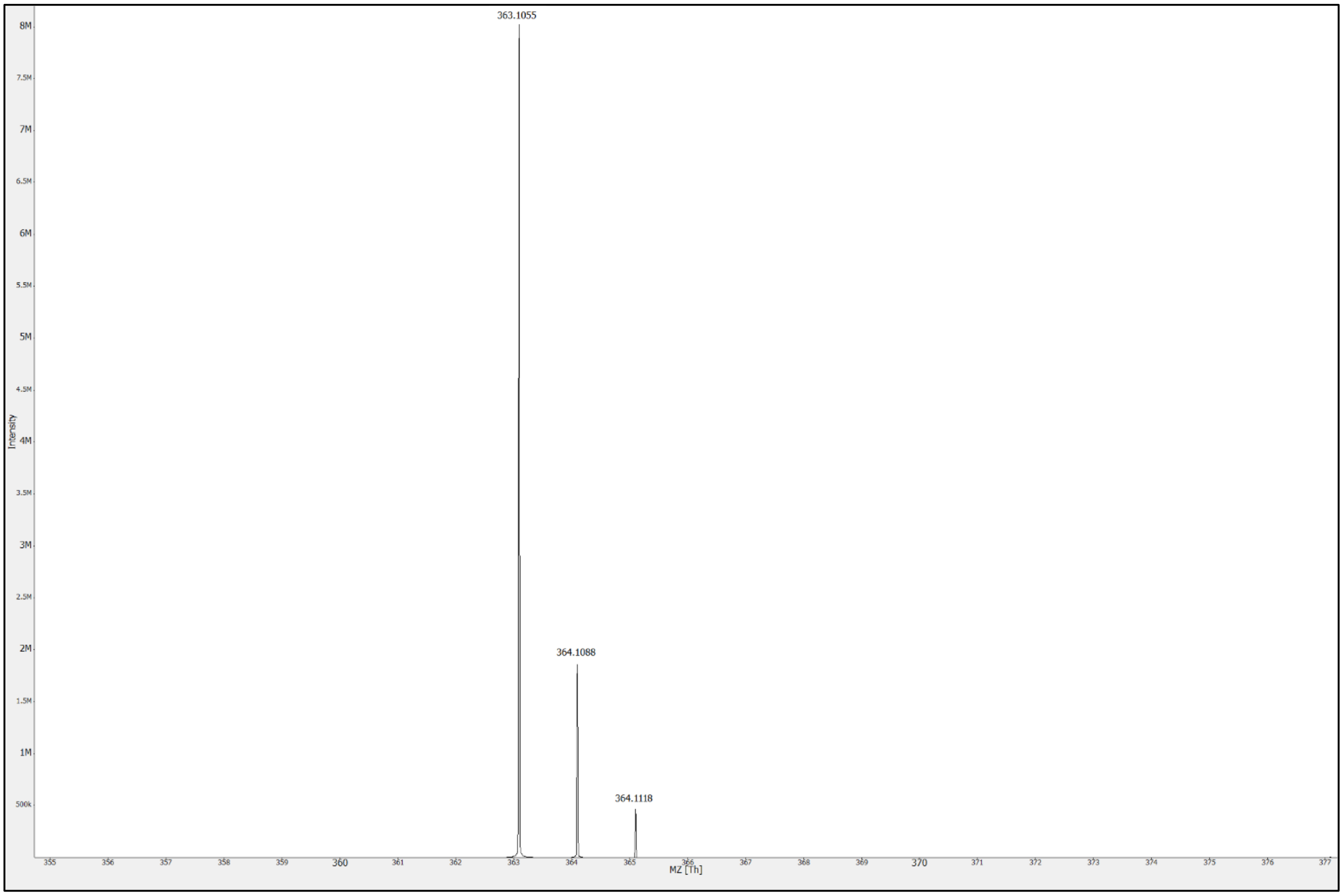

Figure S2. ^{1}H NMR spectrum of compound **1** (CD_3OD).

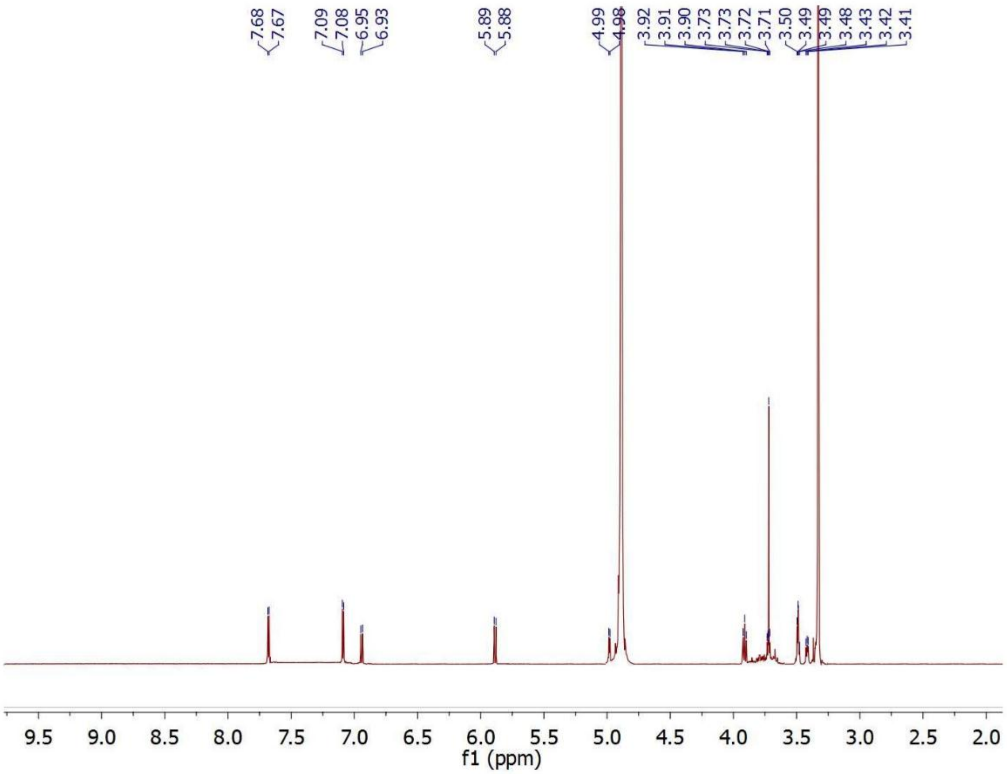

Figure S3. Expansion of ^{1}H NMR spectrum of compound **1** (CD_3OD).

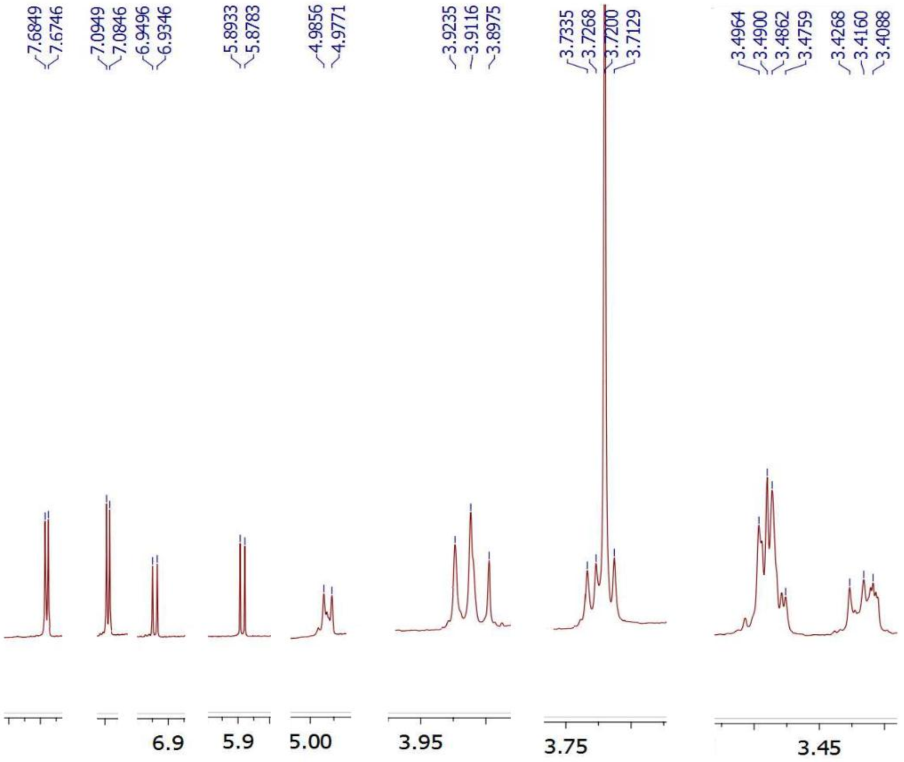

Figure S4. ^{13}C NMR spectrum of compound **1** (CD_3OD).

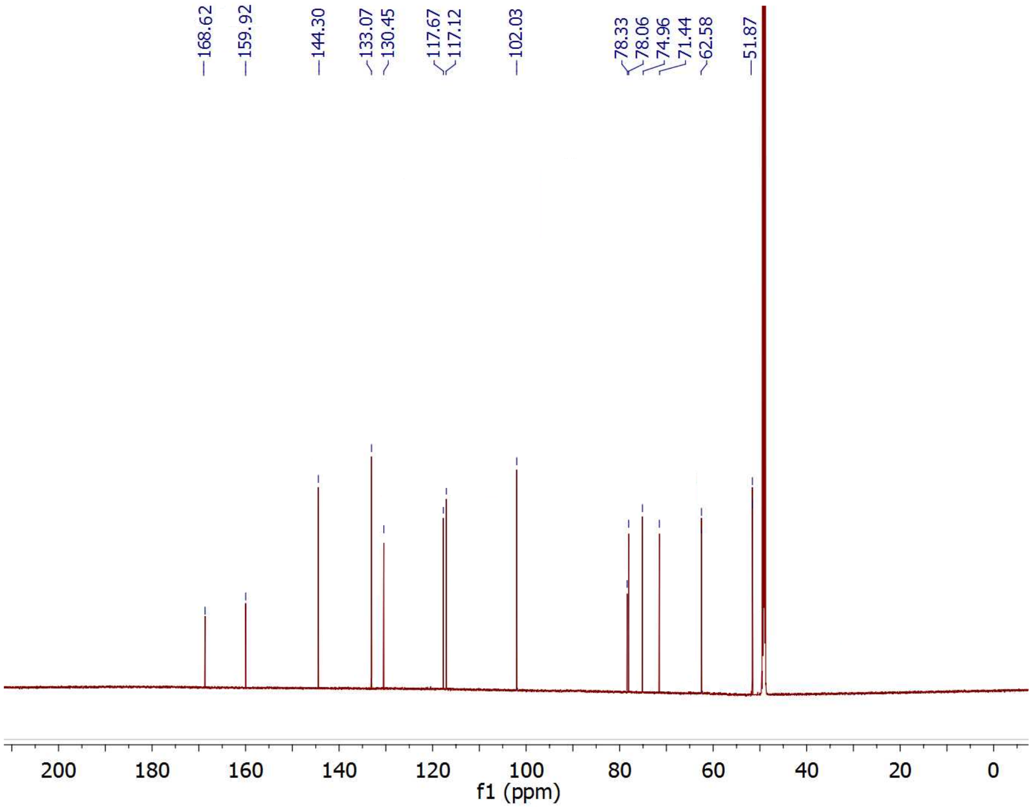

Figure S5. DEPT spectrum of compound **1** (CD_3OD).

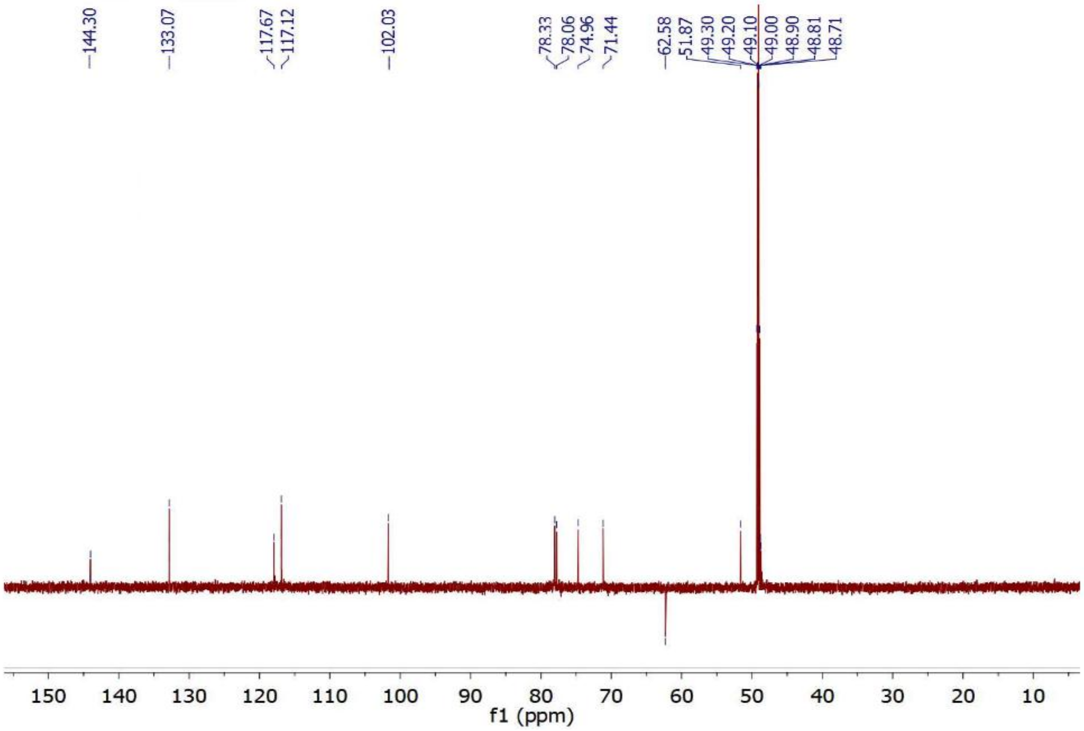

Figure S6. COSY spectrum of compound **1** (CD_3OD).

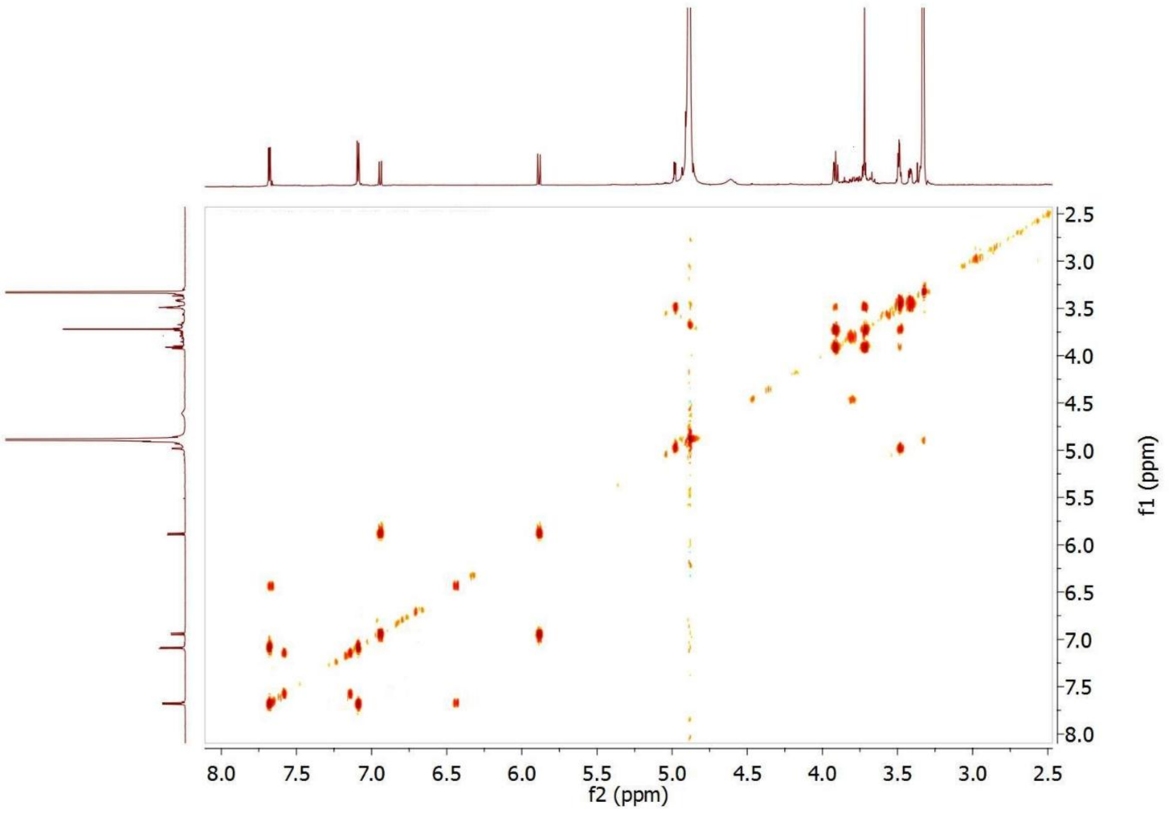

Figure S7. Multiplicity-edited HSQC spectrum of compound **1** (CD_3OD).

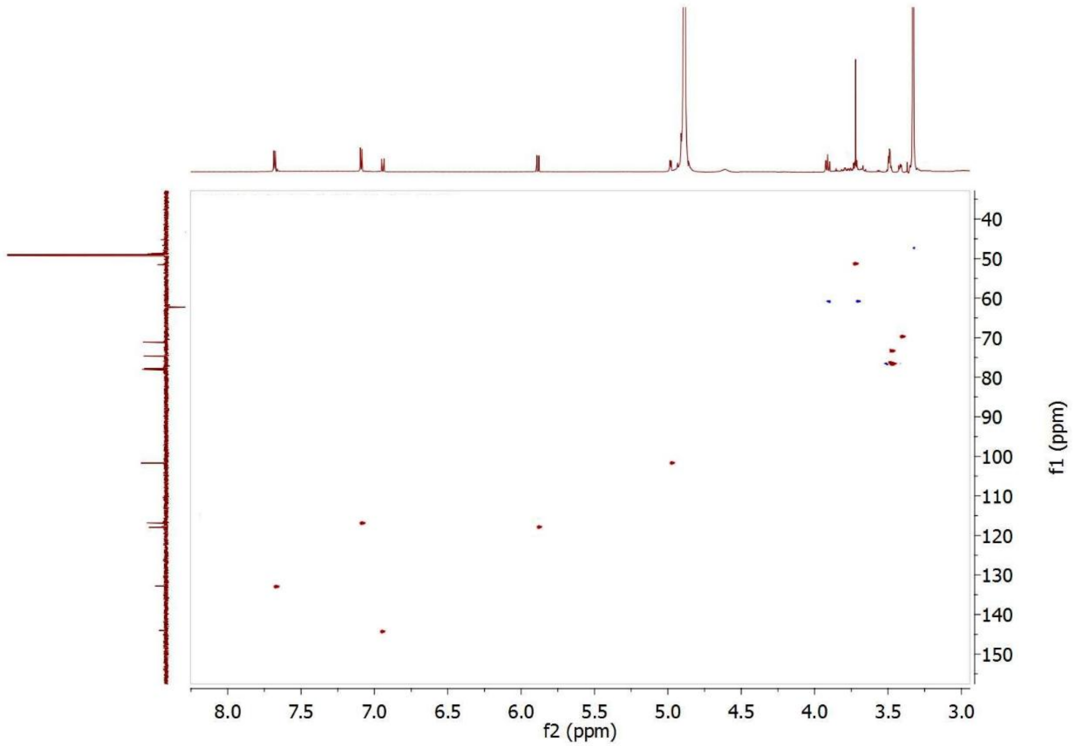

Figure S8. ^{1}H-^{13}C HMBC spectrum of compound **1** (CD_3OD).

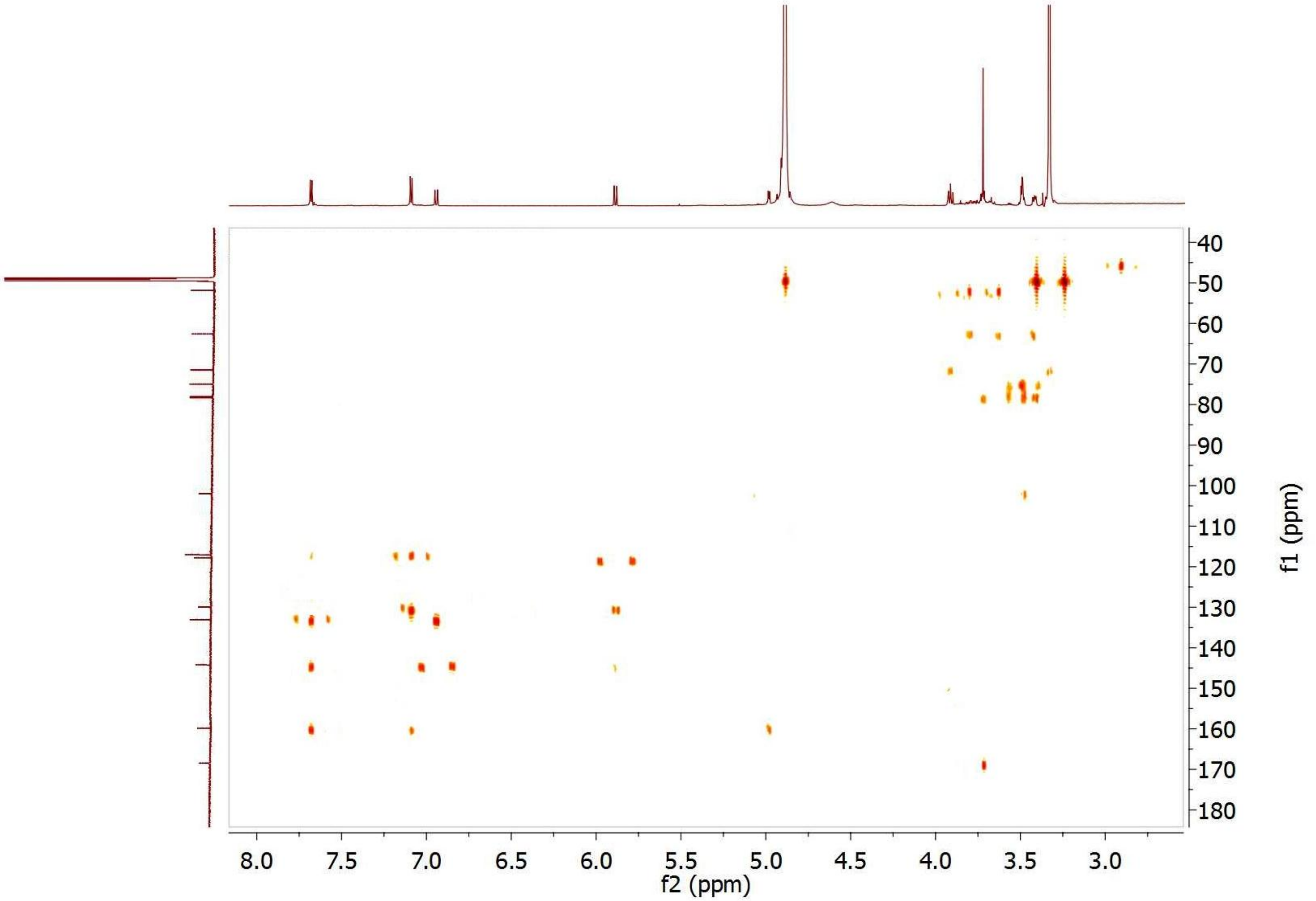

Figure S9. (+)-HRESIMS spectrum of compound **2**.

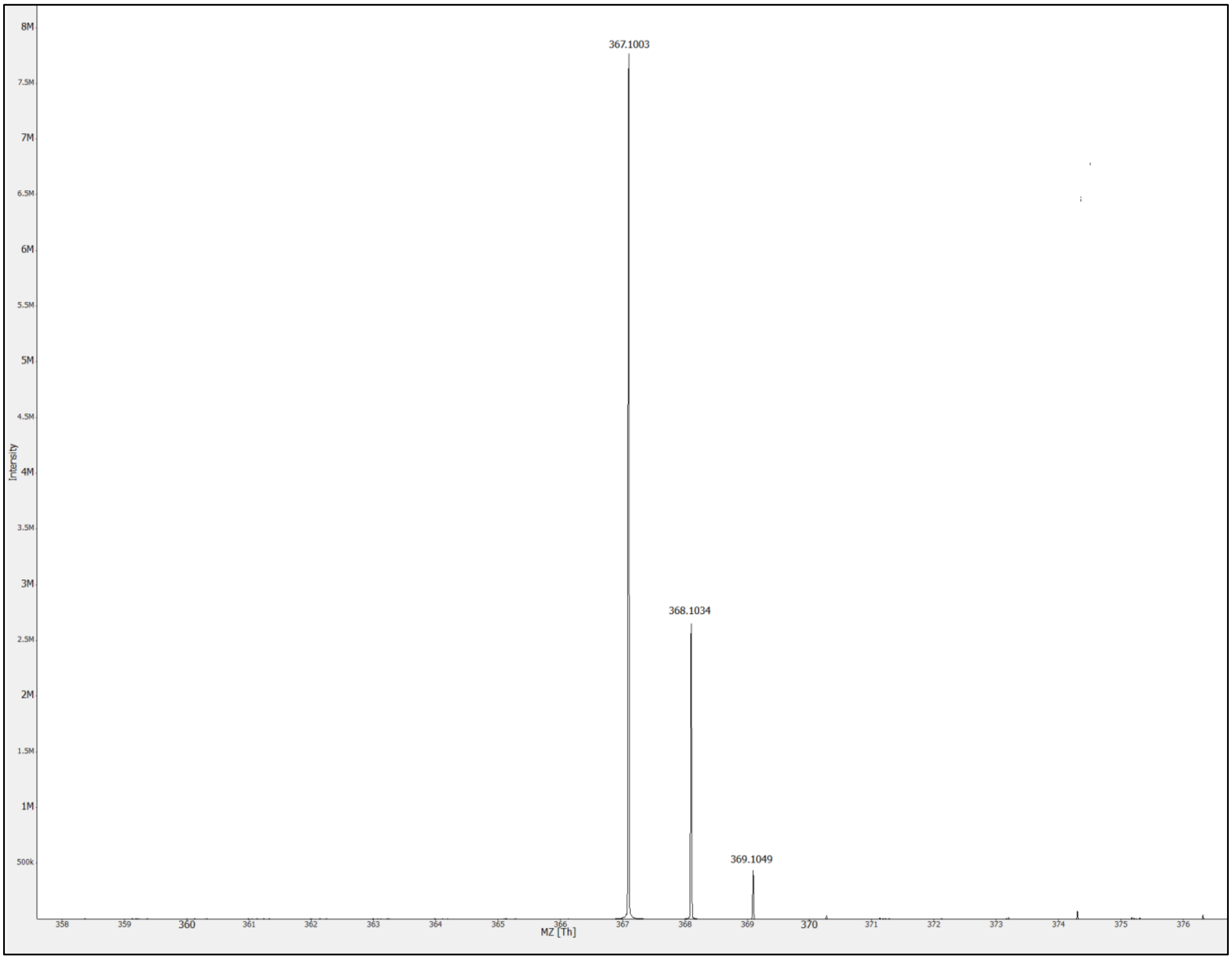

Figure S10. ^{1}H NMR spectrum of compound **2** (CD_3OD).

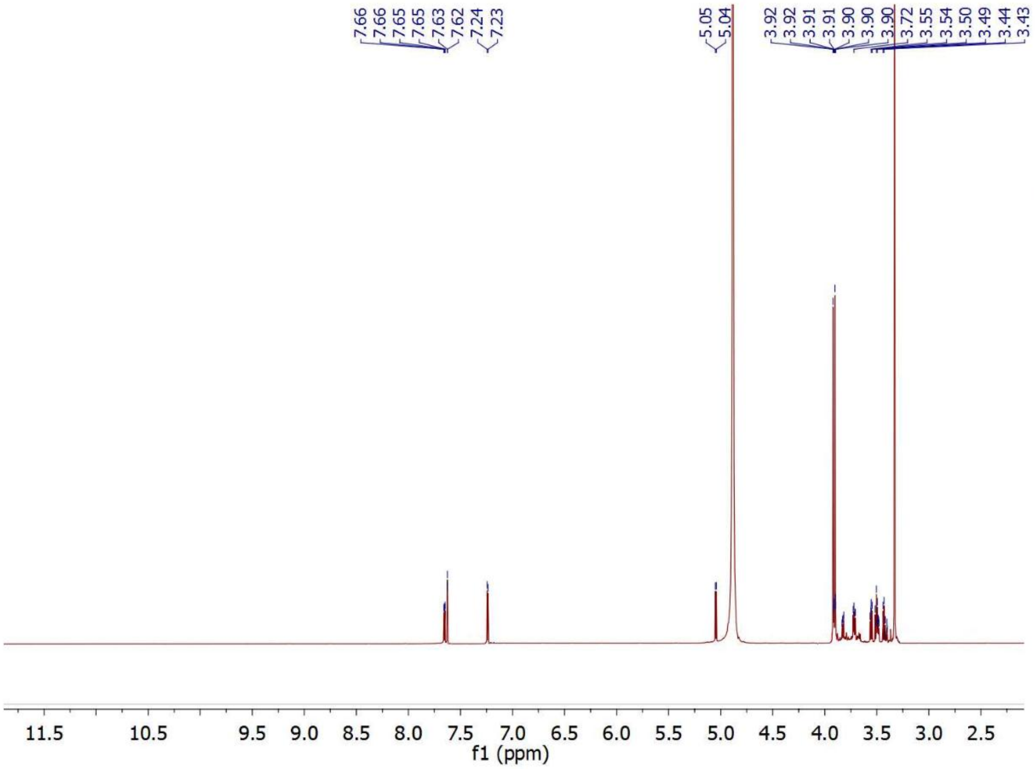

Figure S11. Expansion of ^{1}H NMR spectrum of compound **2** (CD_3OD).

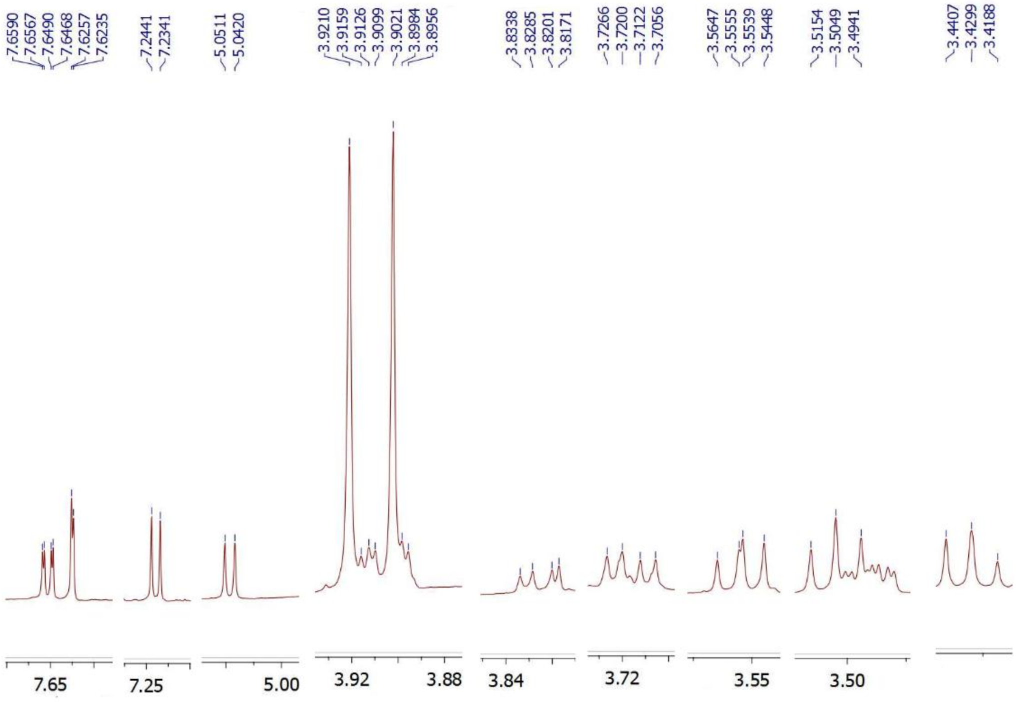

Figure S12. ^{13}C NMR spectrum of compound **2** (CD_3OD).

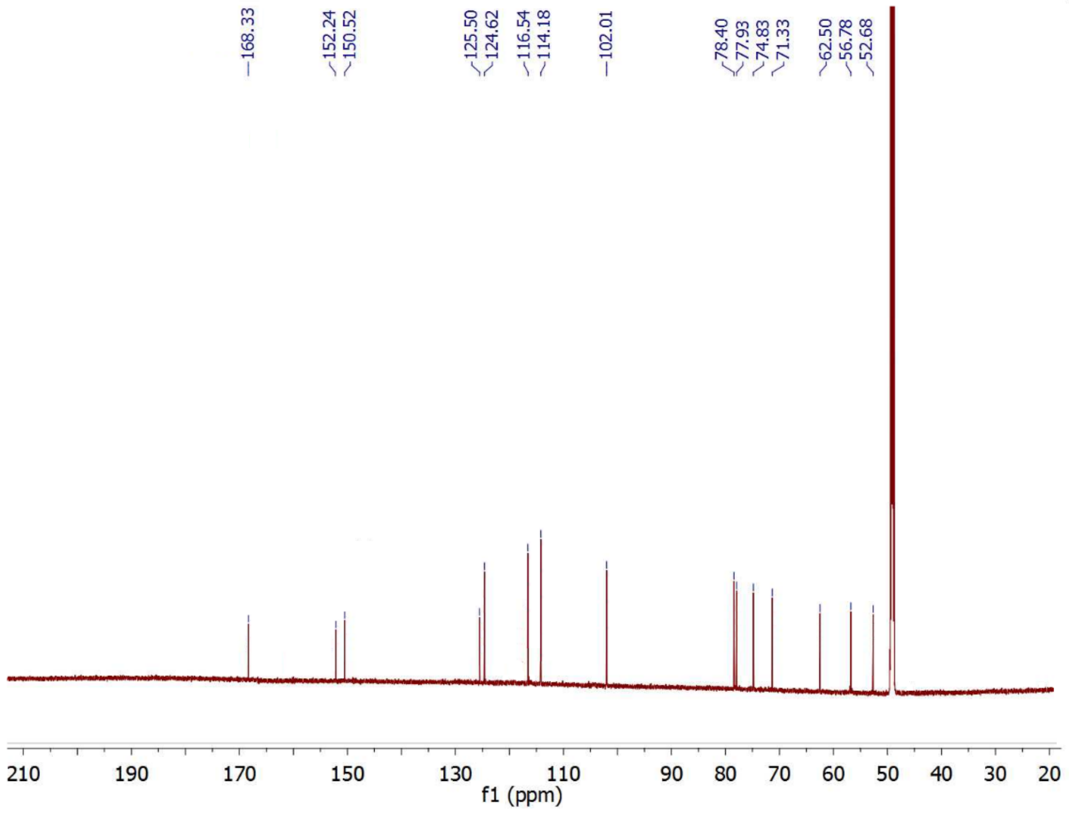

Figure S13. DEPT spectrum of compound **2** (CD_3OD).

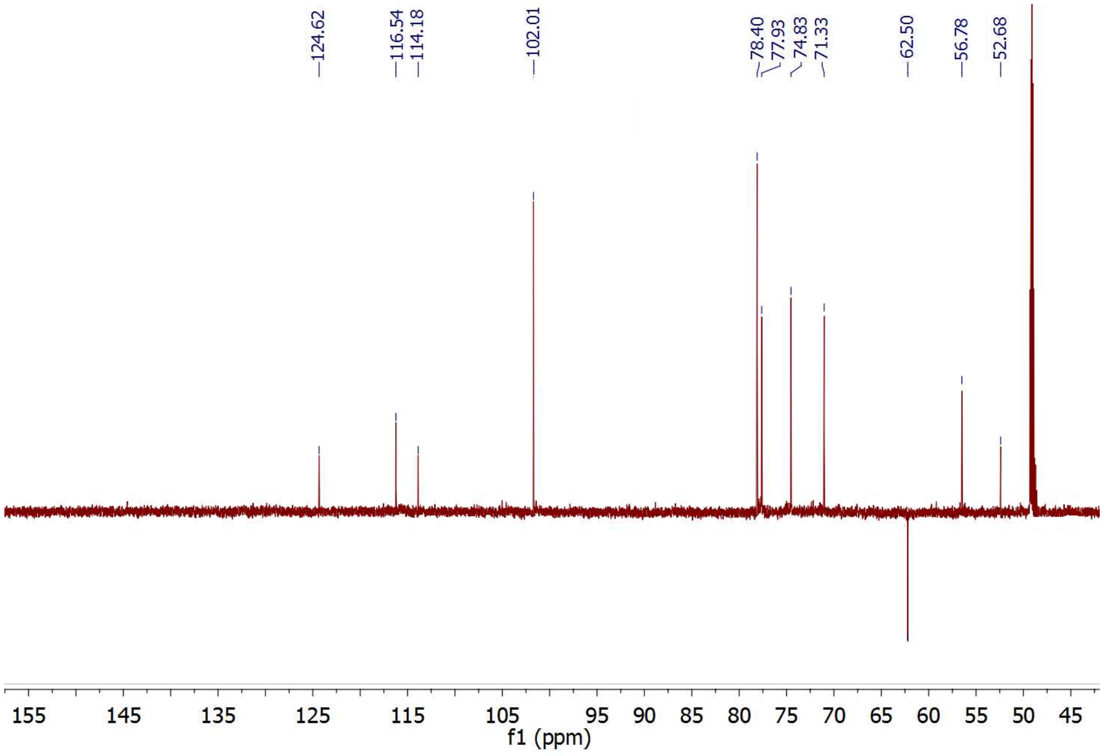

Figure S14. COSY spectrum of compound **2** (CD_3OD).

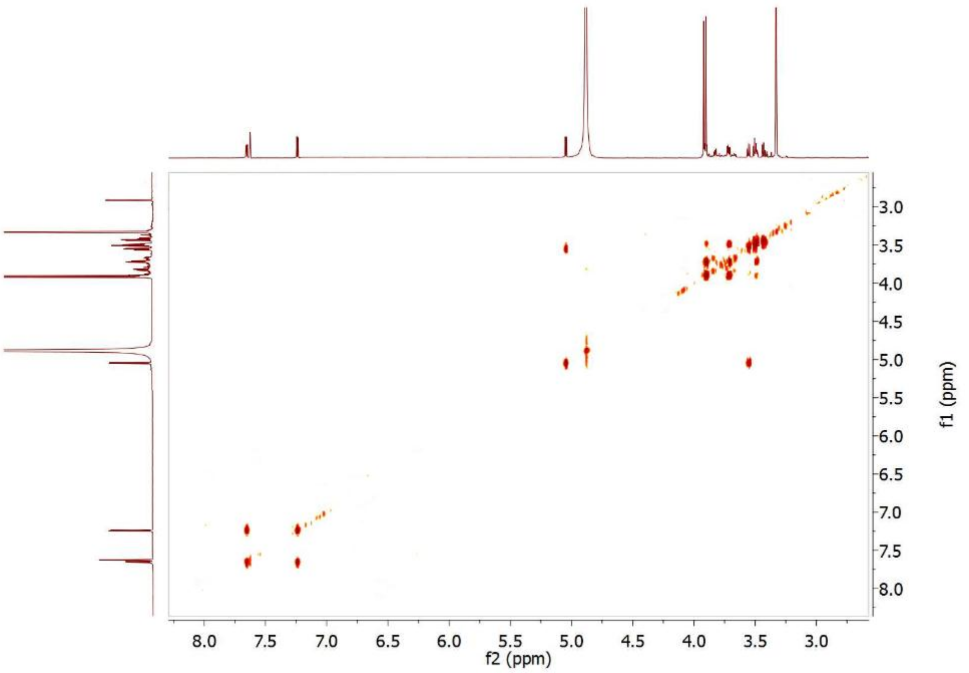

Figure S15. Multiplicity-edited HSQC spectrum of compound **2** (CD_3OD).

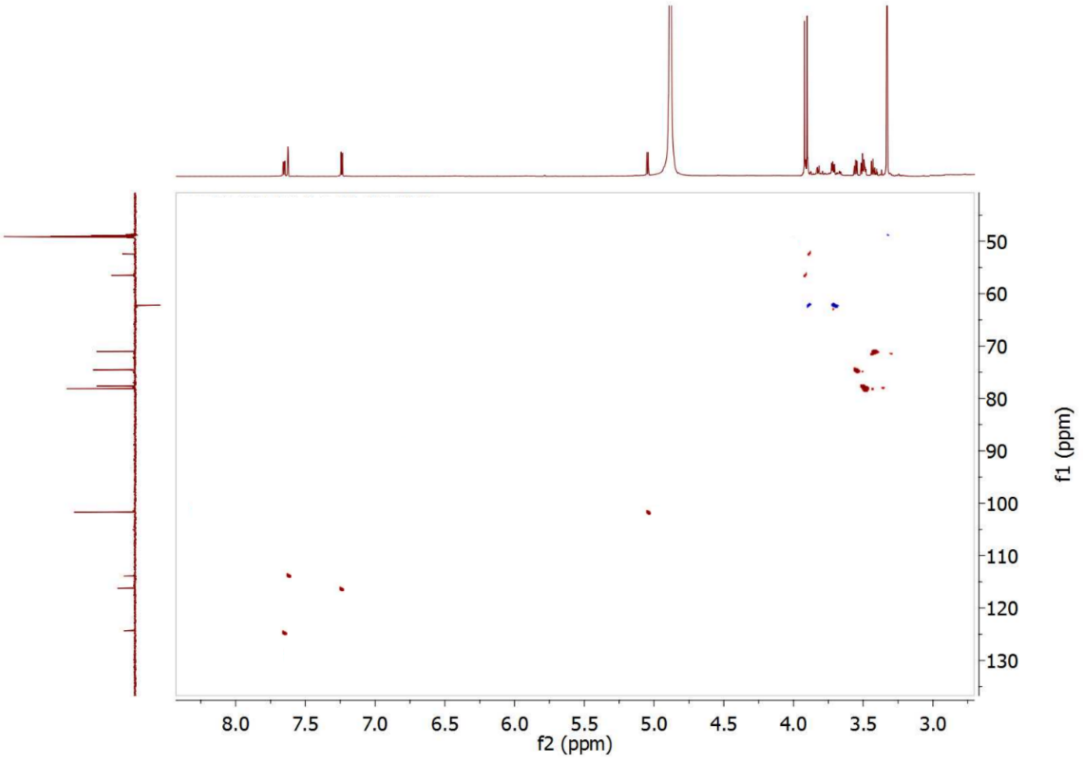

Figure S16. ^{1}H-^{13}C HMBC spectrum of compound **2** (CD_3OD).

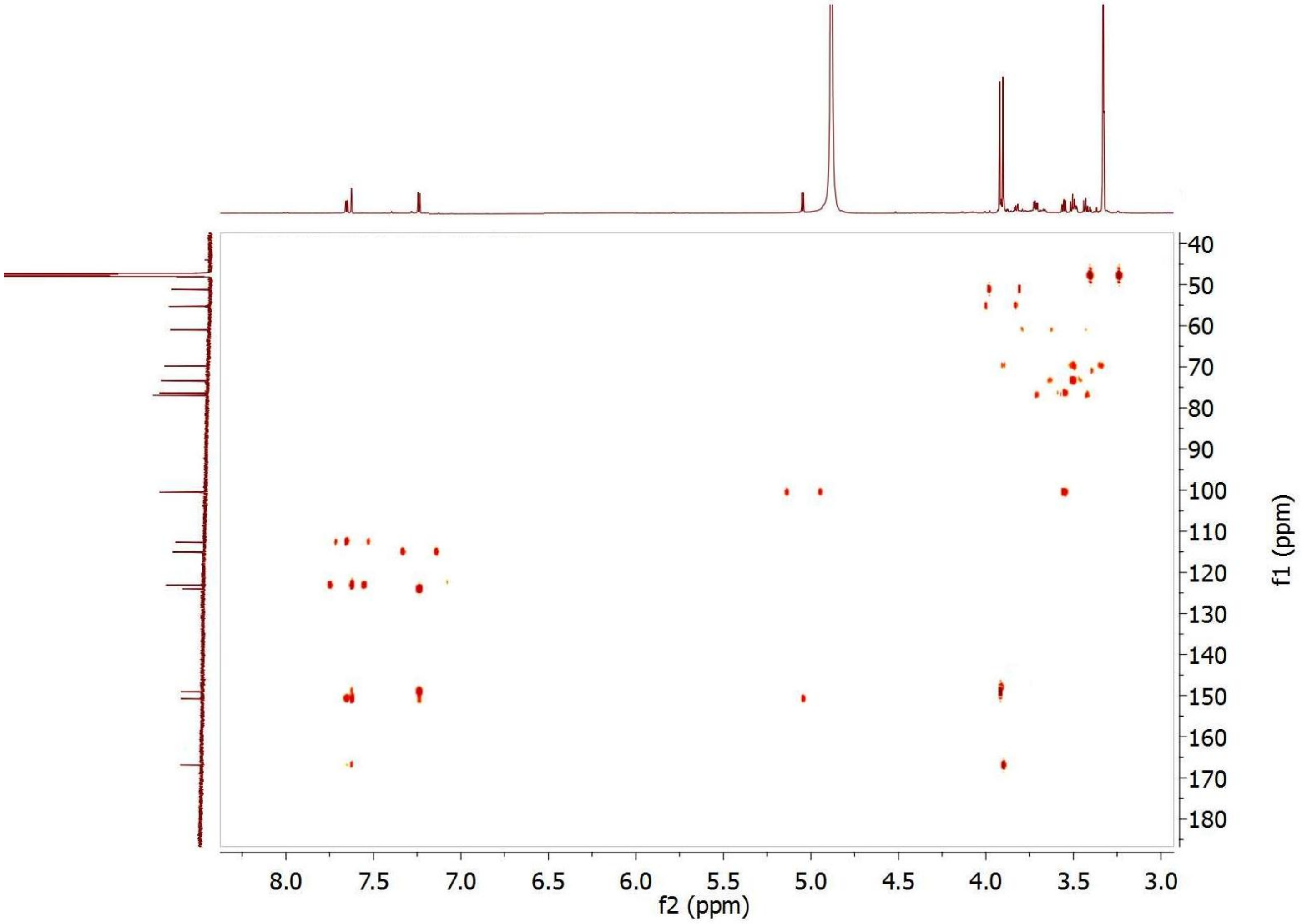

Figure S17. (+)-HRESIMS spectrum of compound **3**.

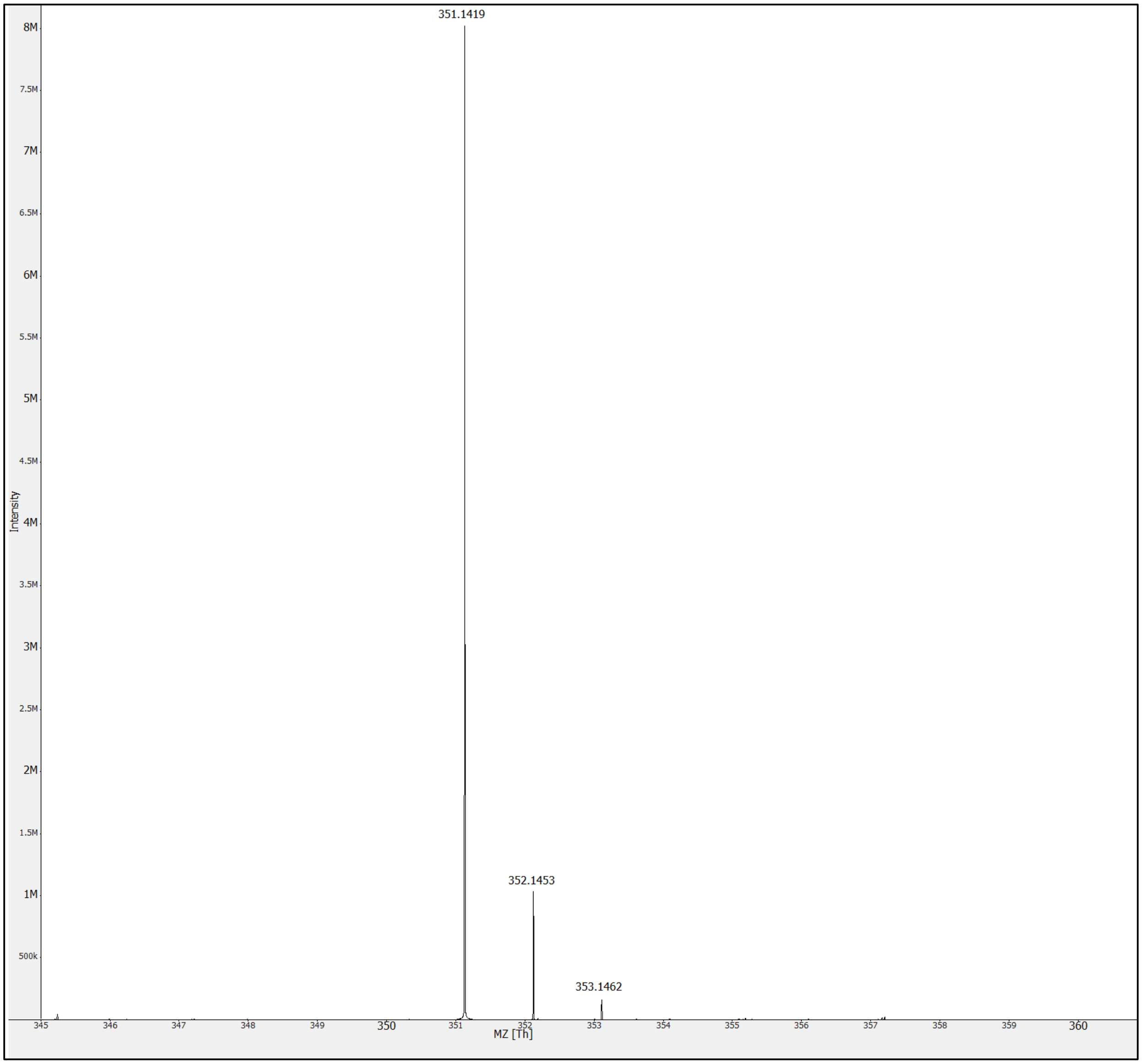

Figure S18. ^{1}H NMR spectrum of compound **3** (CD_3OD).

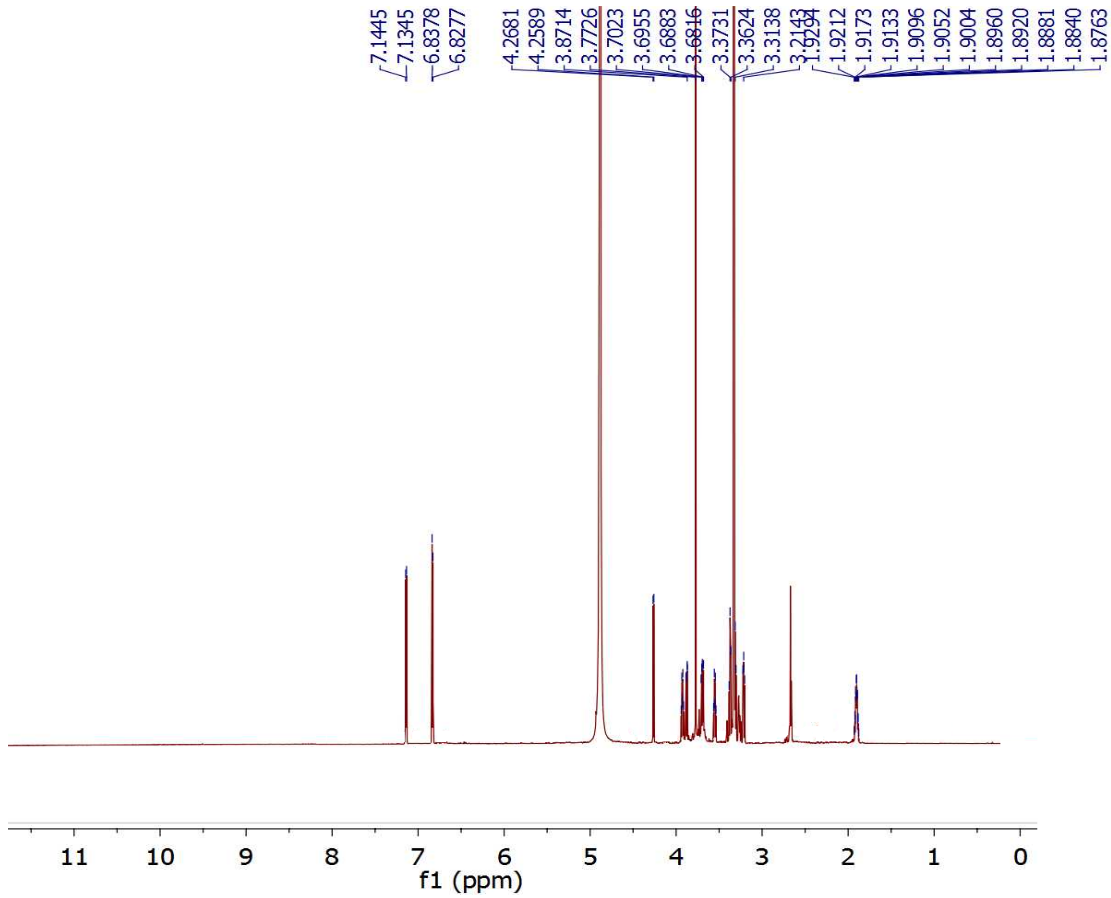

Figure S19. Expansion of ^{1}H NMR spectrum of compound **3** (CD_3OD).

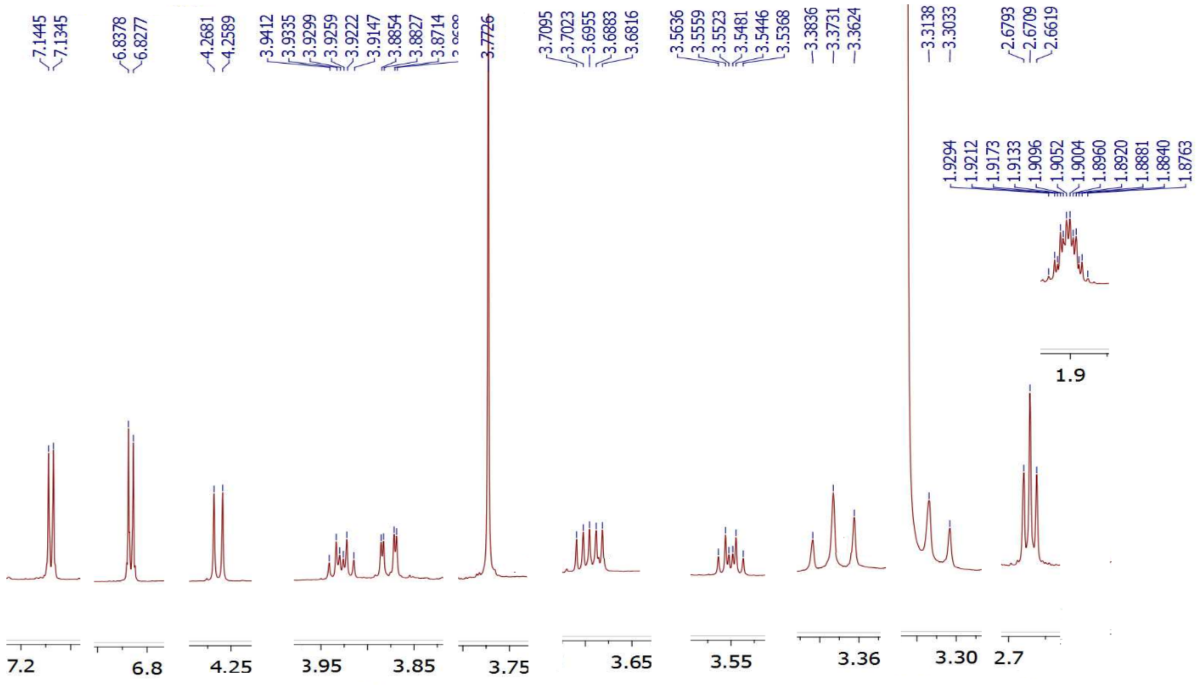

Figure S20. ^{13}C NMR spectrum of compound **3** (CD_3OD).

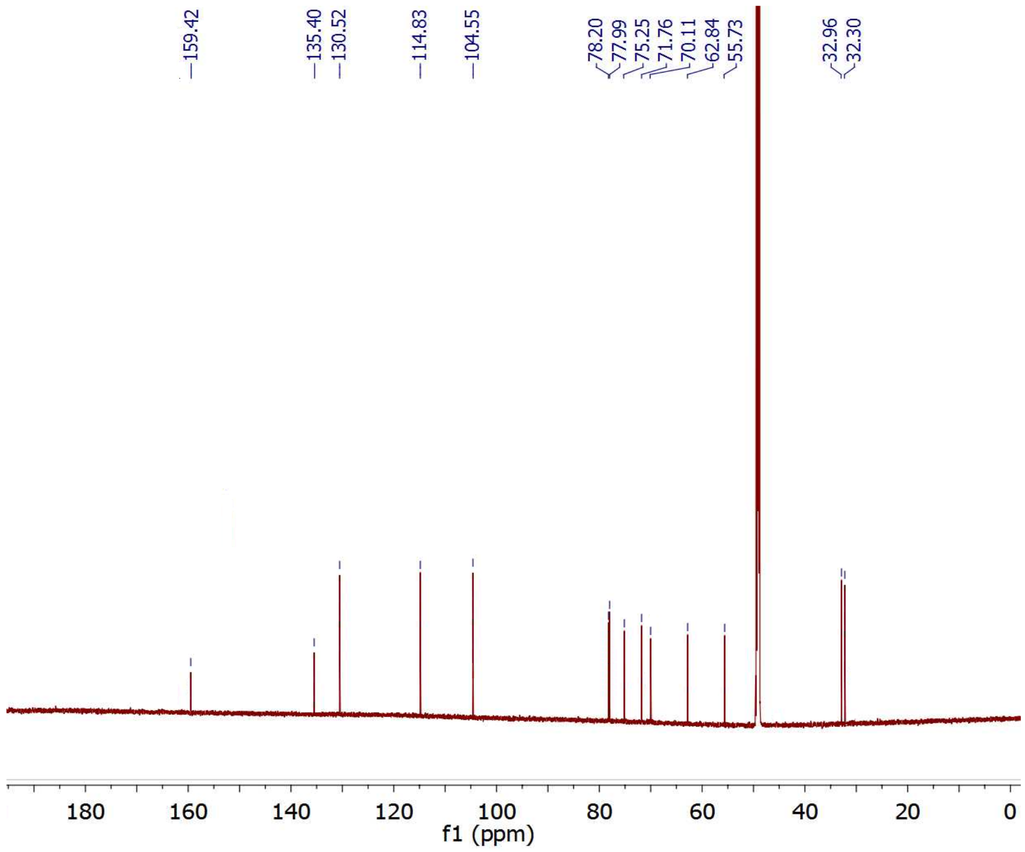

Figure S21. DEPT spectrum of compound **3** (CD_3OD).

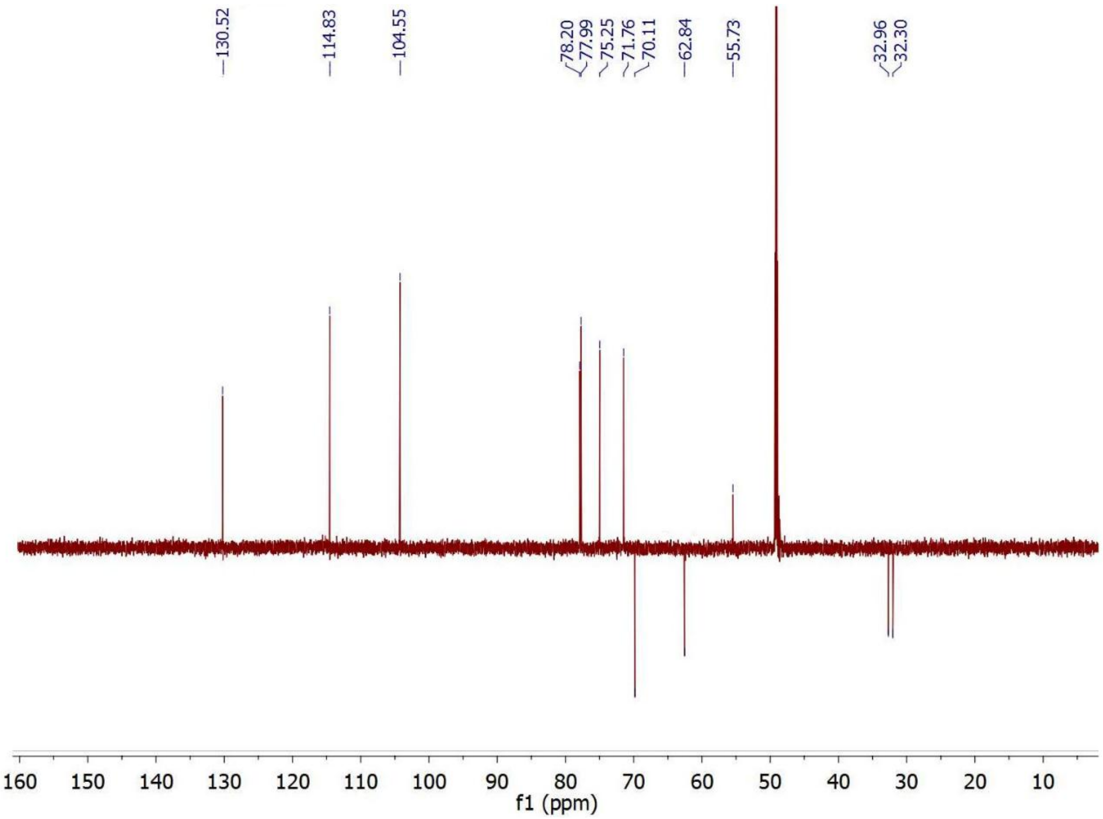

Figure S22. COSY spectrum of compound **3** (CD_3OD).

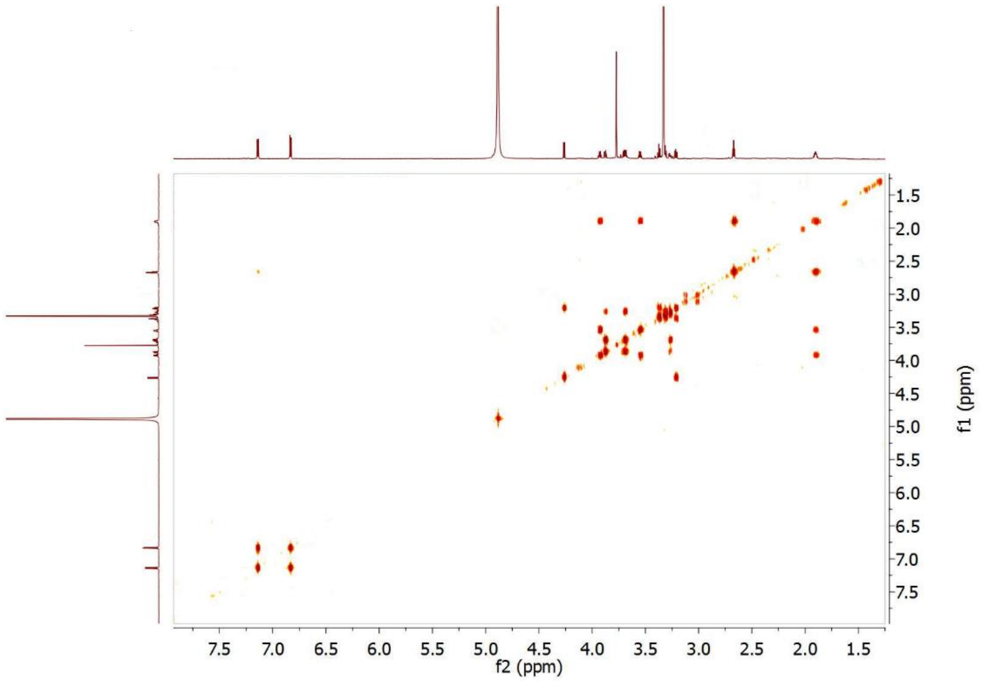

Figure S23. Multiplicity-edited HSQC spectrum of compound **3** (CD_3OD).

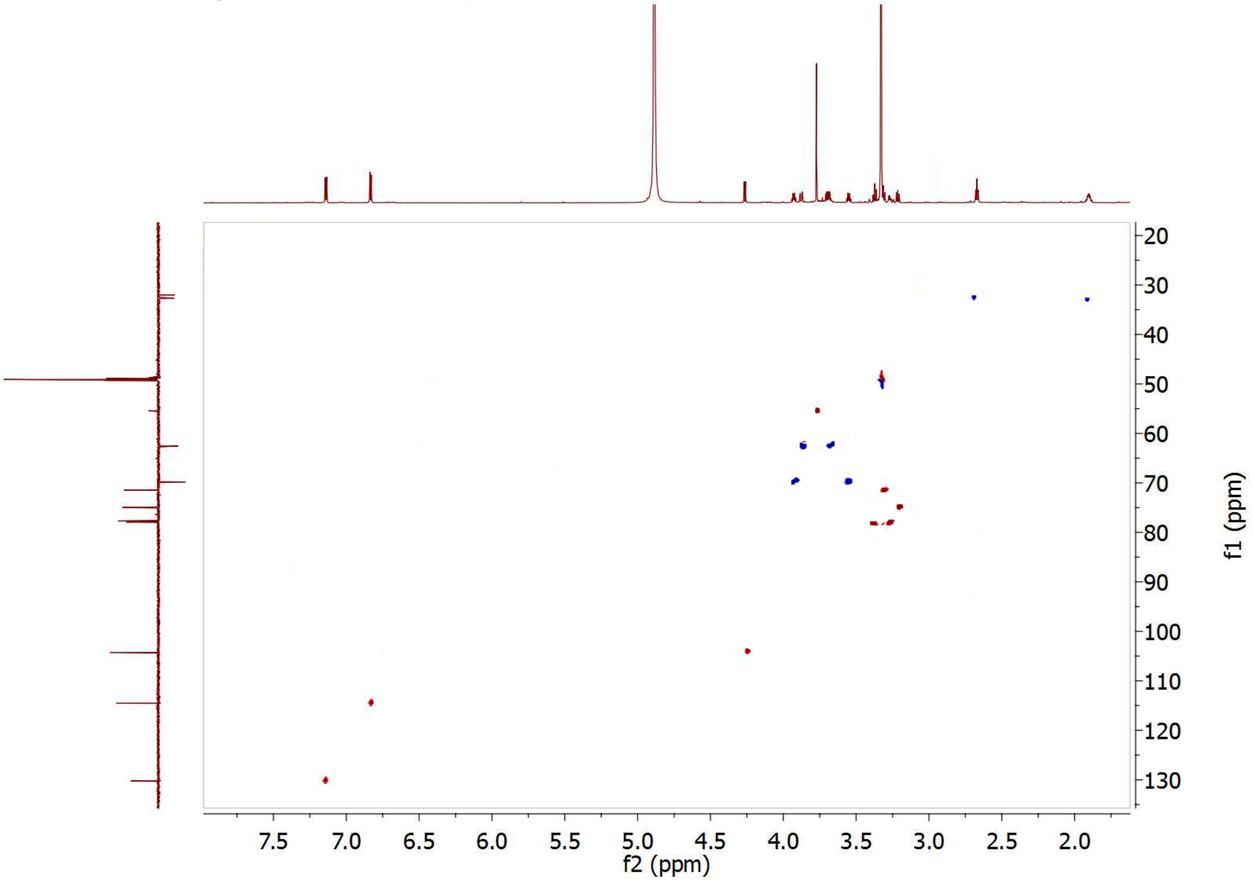

Figure S24. ^{1}H-^{13}C HMBC spectrum of compound **3** (CD_3OD).

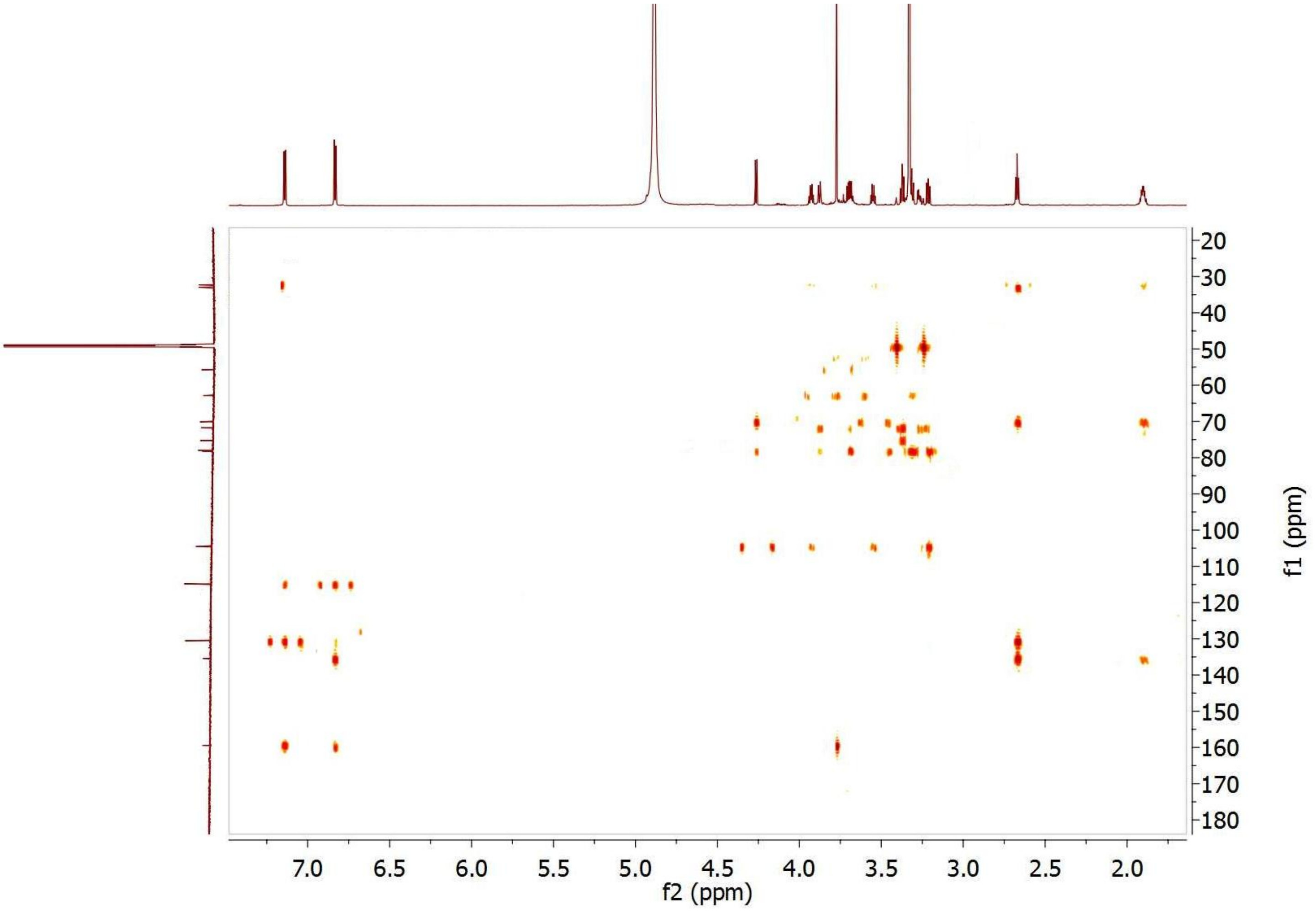

Figure S25. (+)-HRESIMS spectrum of compound **4**.

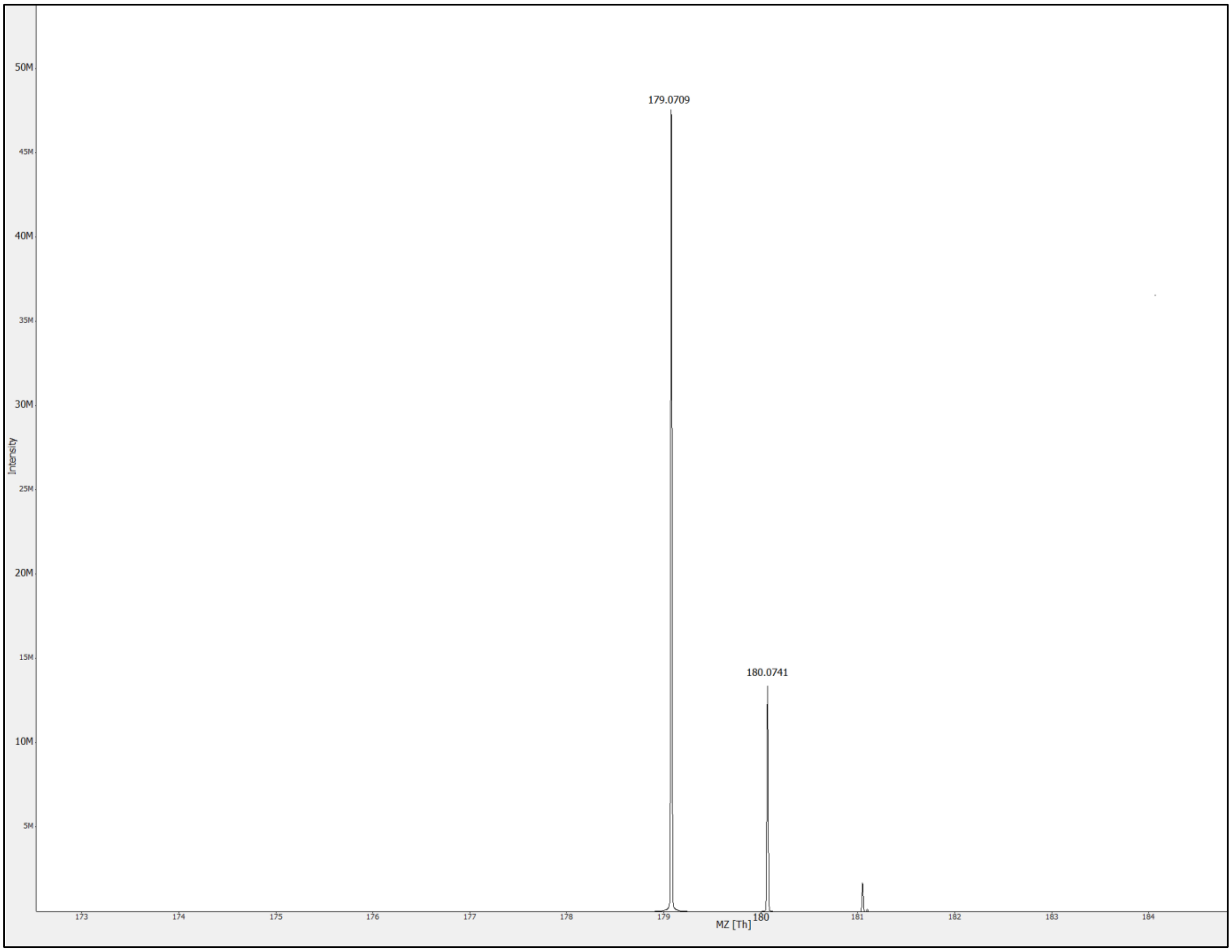

Figure S26. ^{1}H NMR spectrum of compound **4** (CD_3OD).

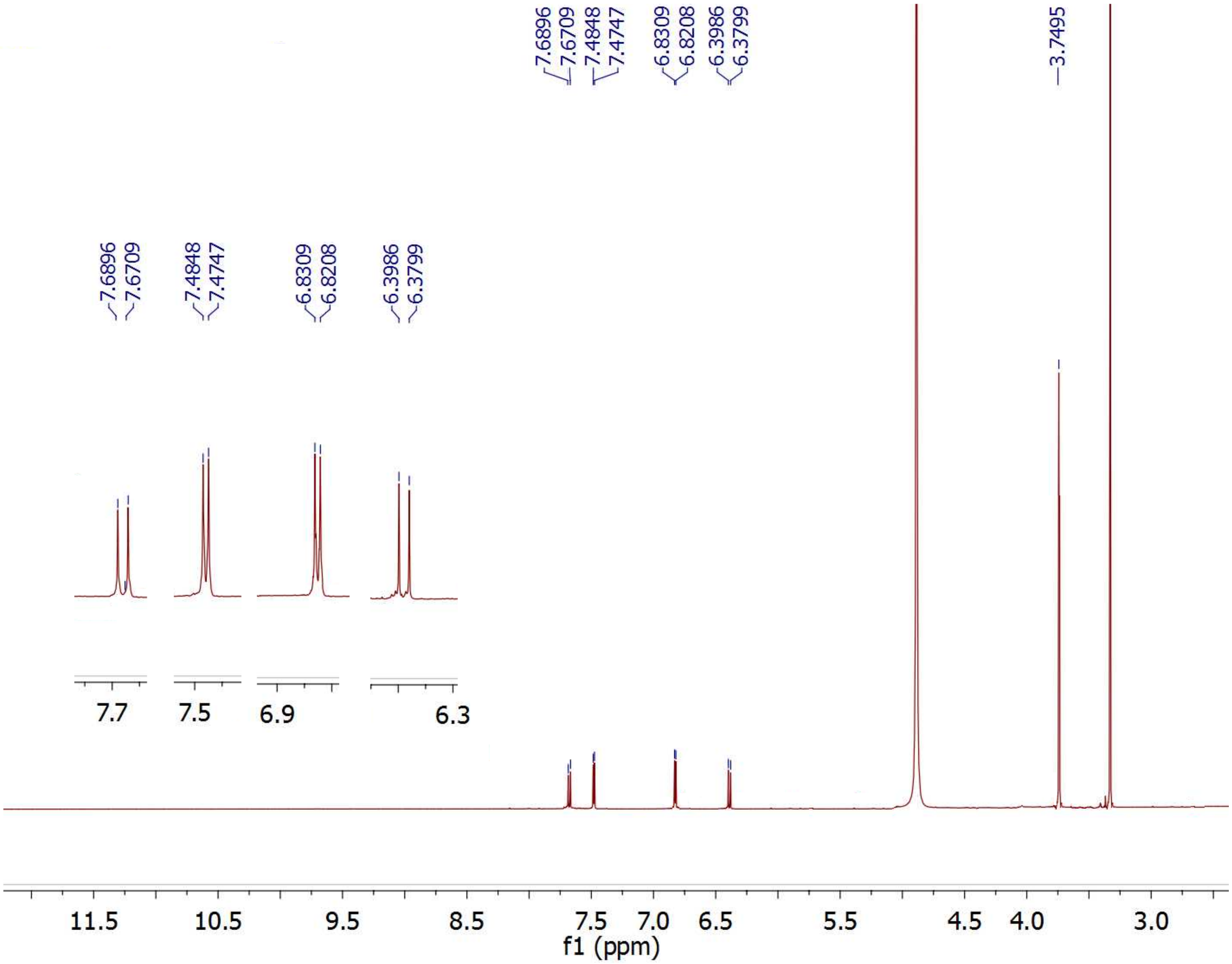

Figure S27. ^{13}C NMR spectrum of compound **4** (CD_3OD).

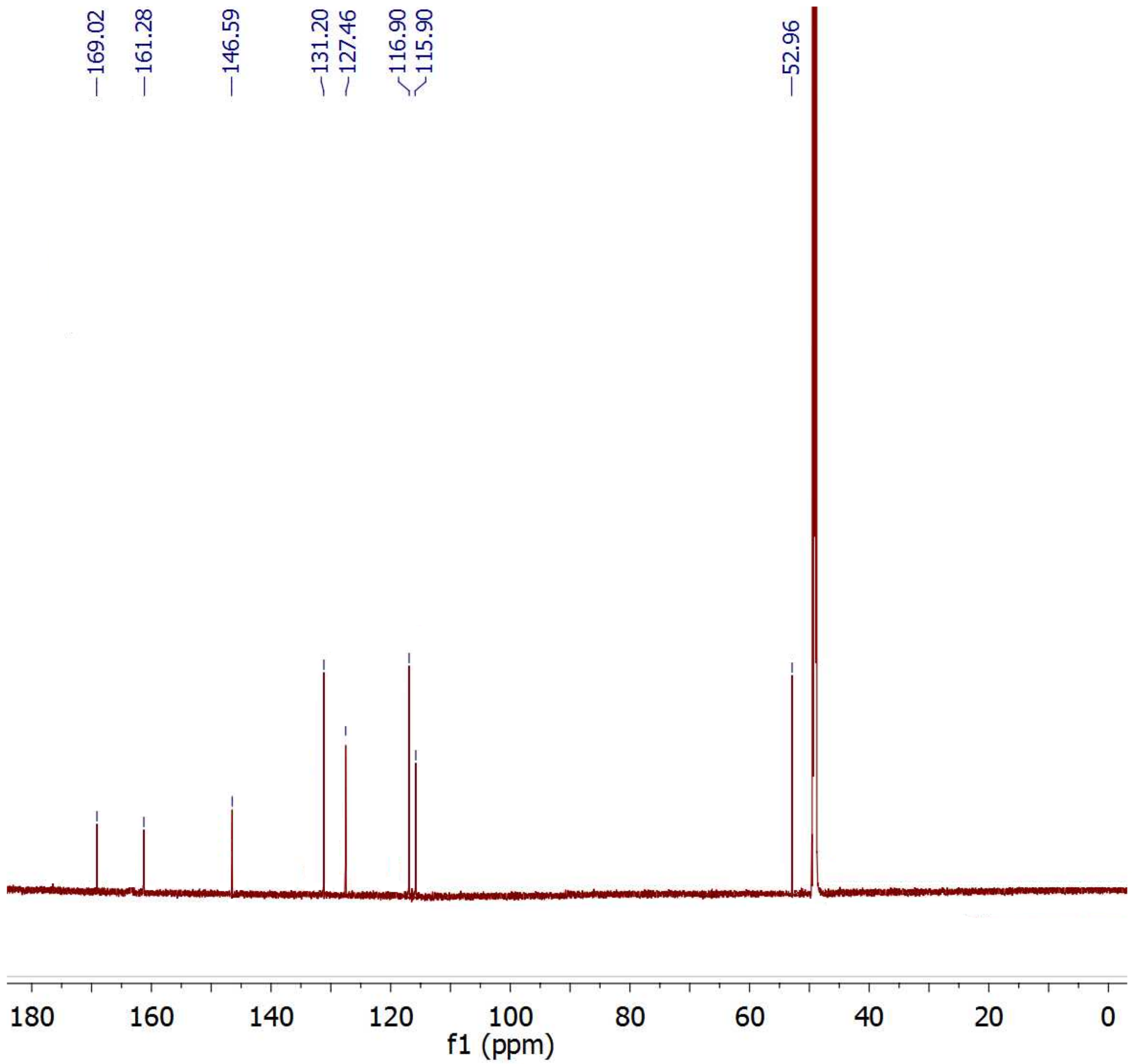

Figure S28. (+)-HRESIMS spectrum of compound **5**.

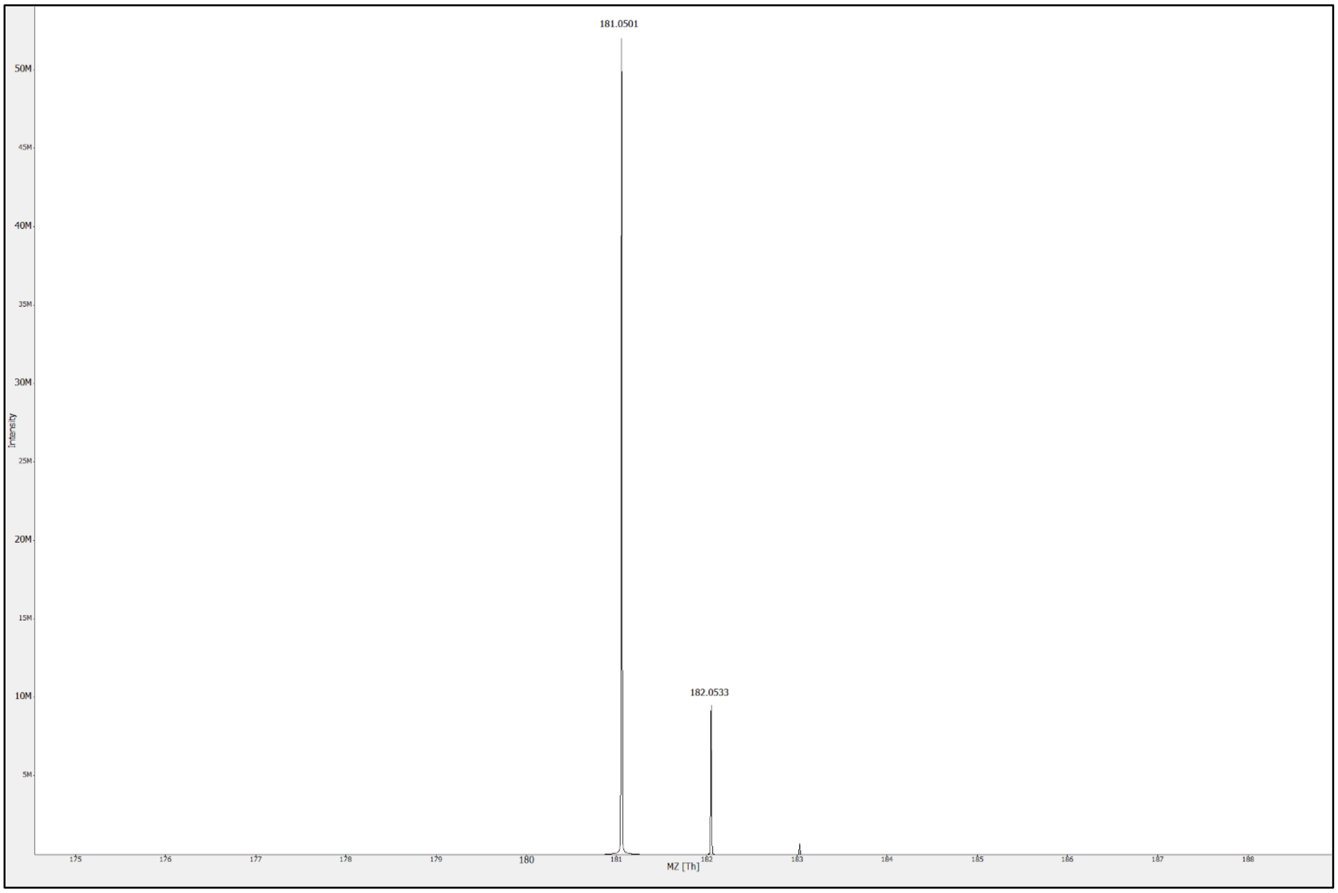

Figure S29. ^{1}H NMR spectrum of compound **5** (CD_3OD).

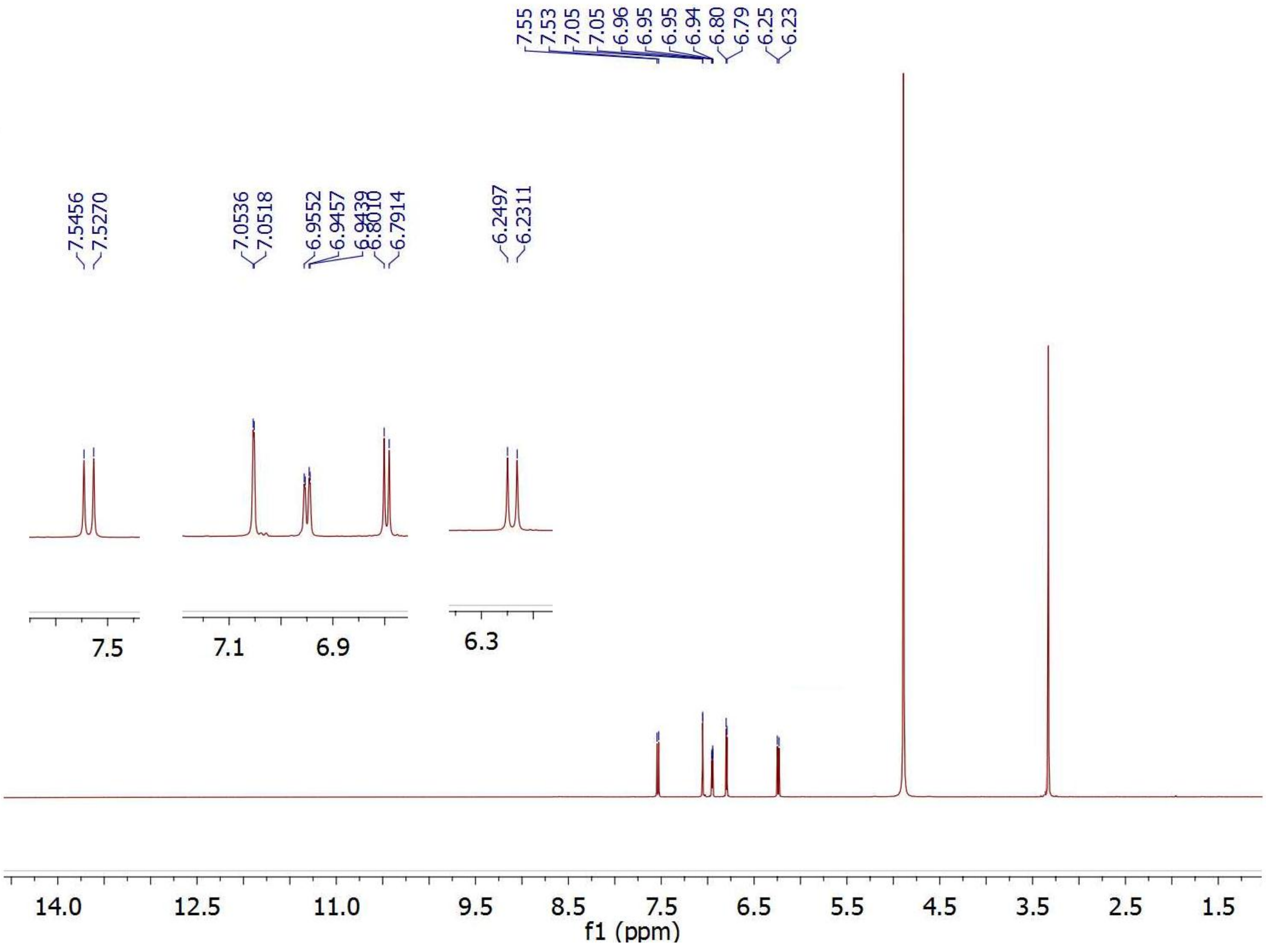

Figure S30. ^{13}C NMR spectrum of compound **5** (CD_3OD).

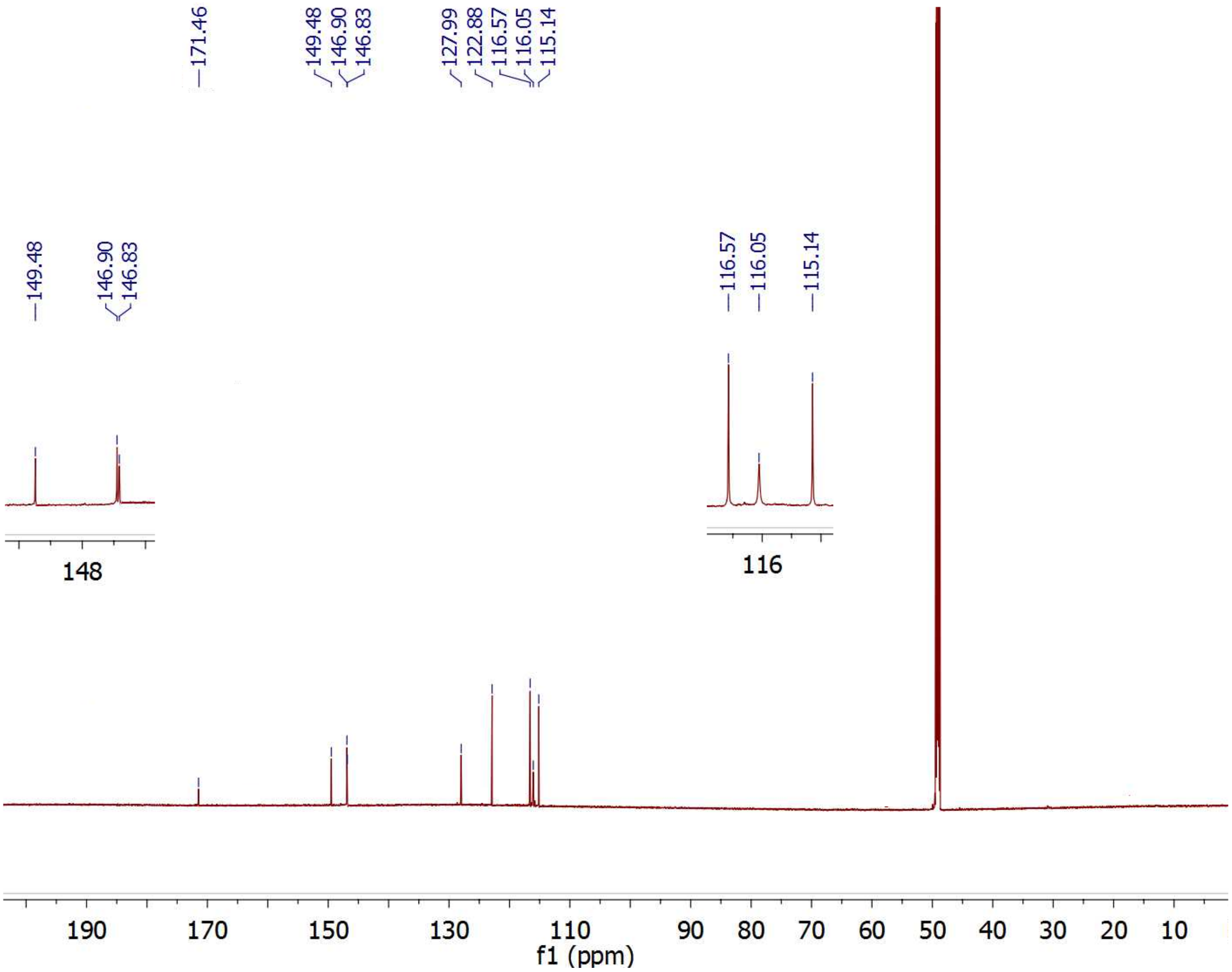

Figure S31. (+)-HRESIMS spectrum of compound **6**.

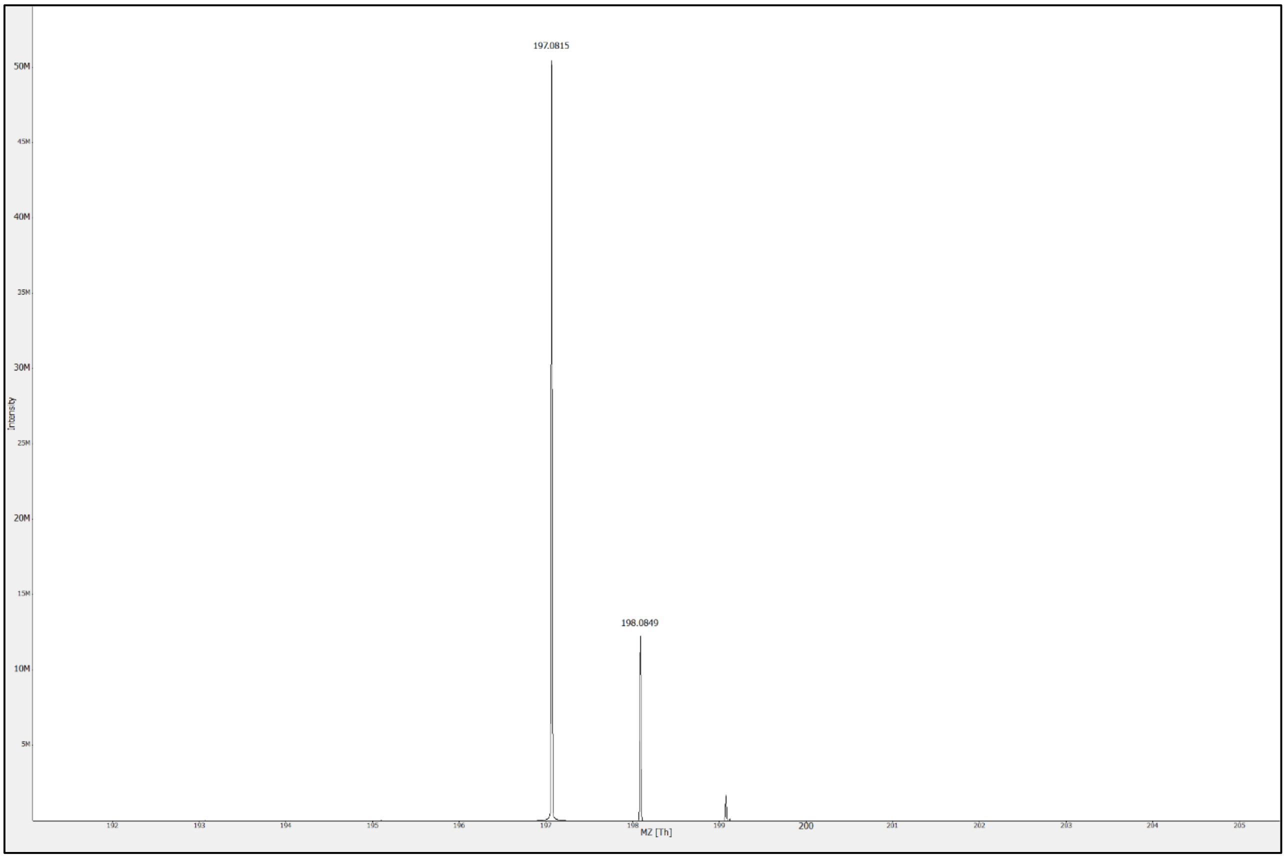

Figure S32. ^{1}H NMR spectrum of compound **6** (CD_3OD).

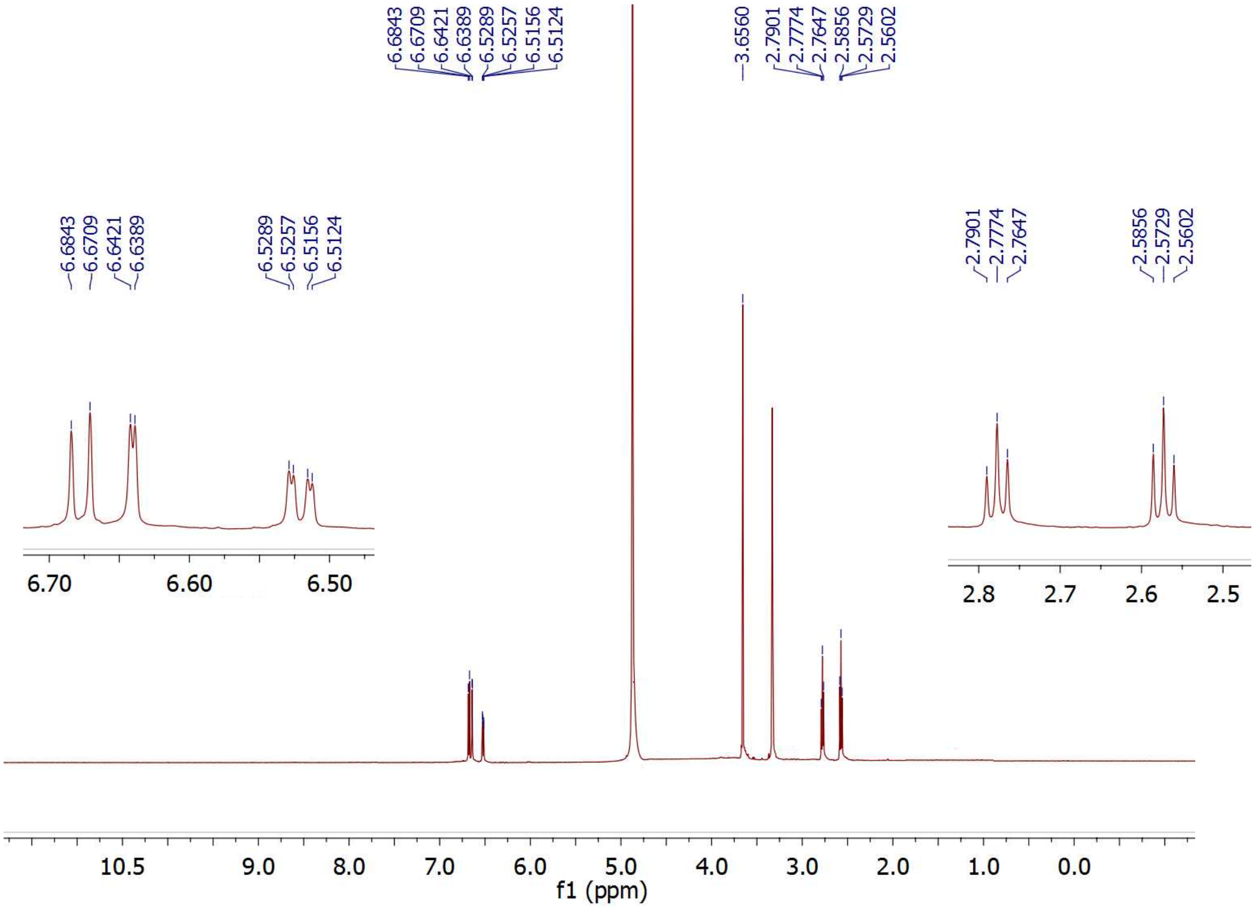

Figure S33. ^{13}C NMR spectrum of compound **6** (CD_3OD).

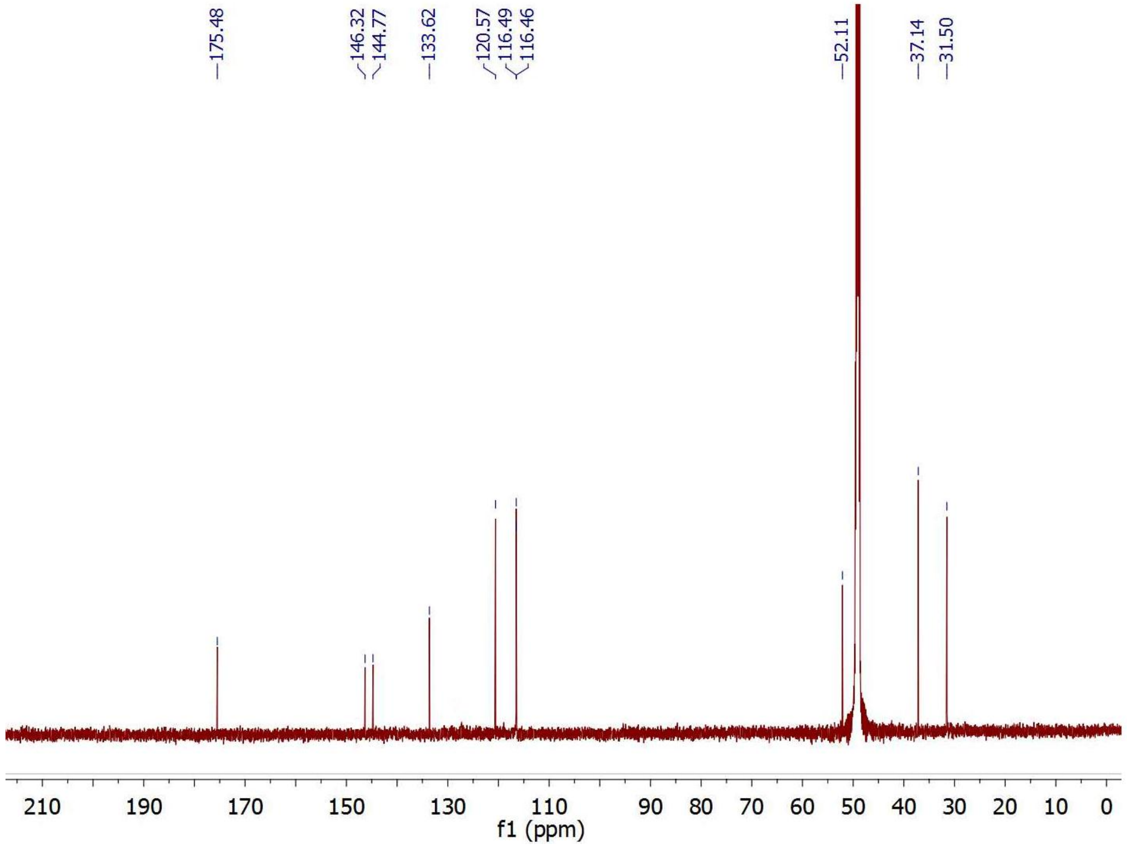

Figure S34. (+)-HRESIMS spectrum of compound **7**.

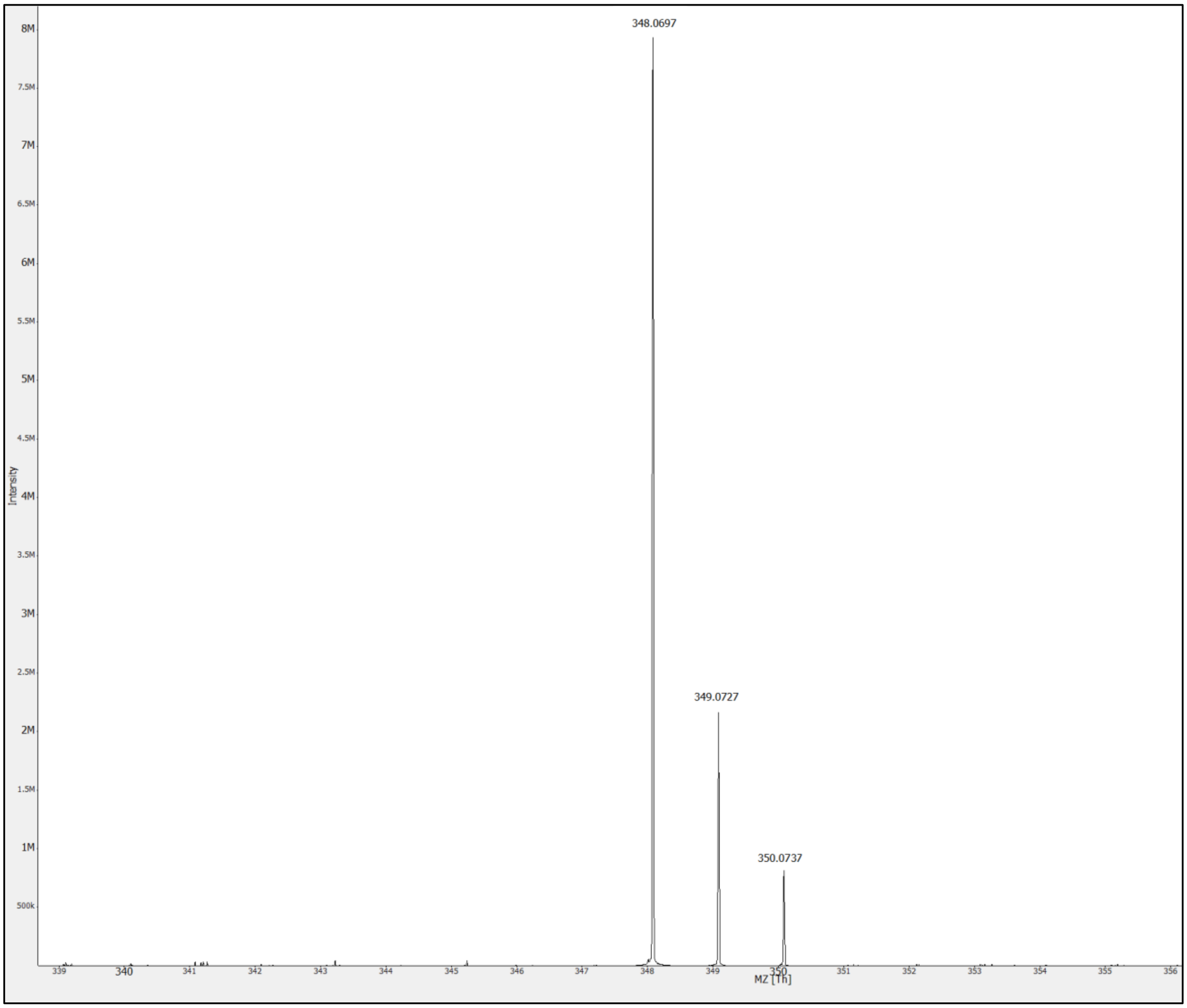

Figure S35. ^{1}H NMR spectrum of compound **7**·(DMSO-d_6).

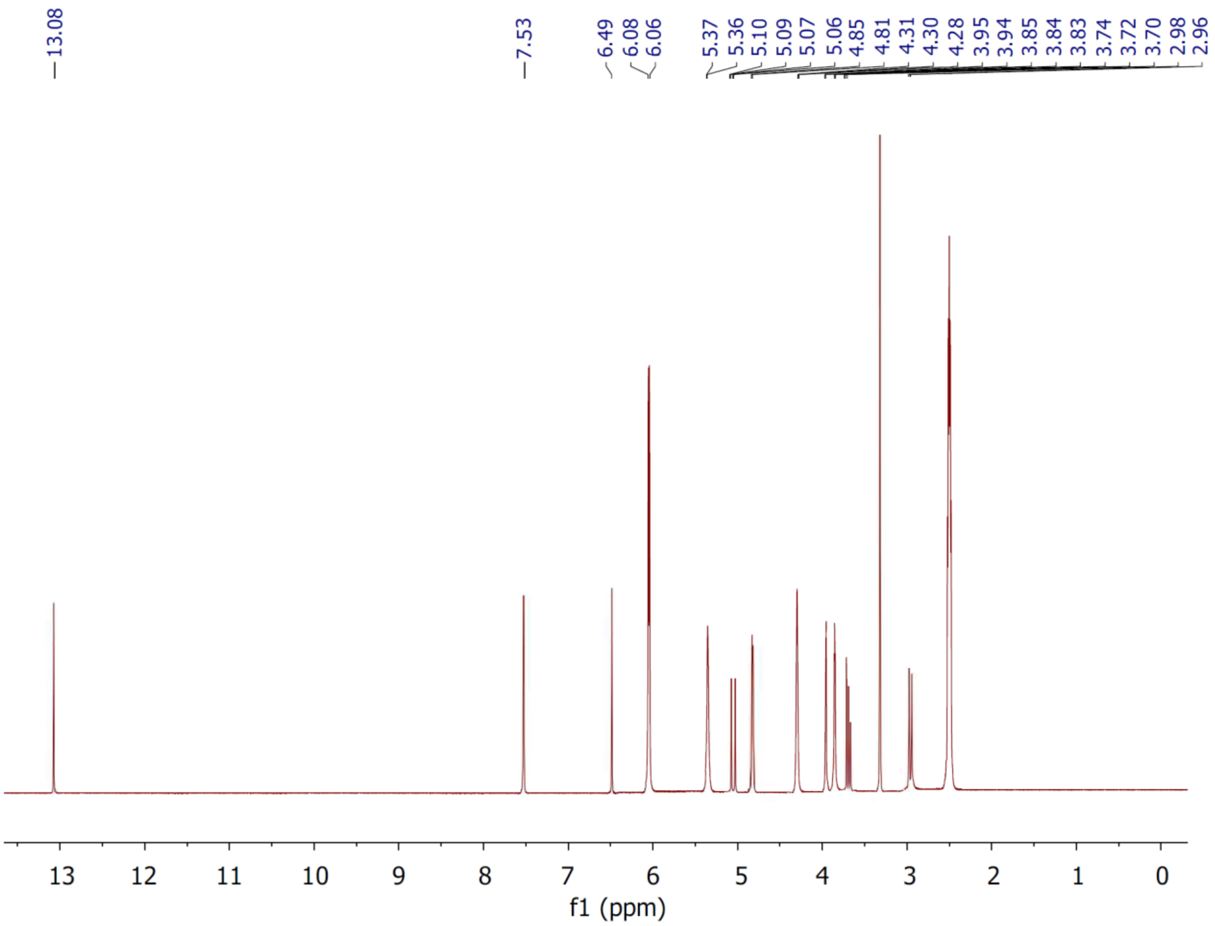

Figure S36. ^{13}C NMR spectrum of compound **7**·(DMSO-d_6).

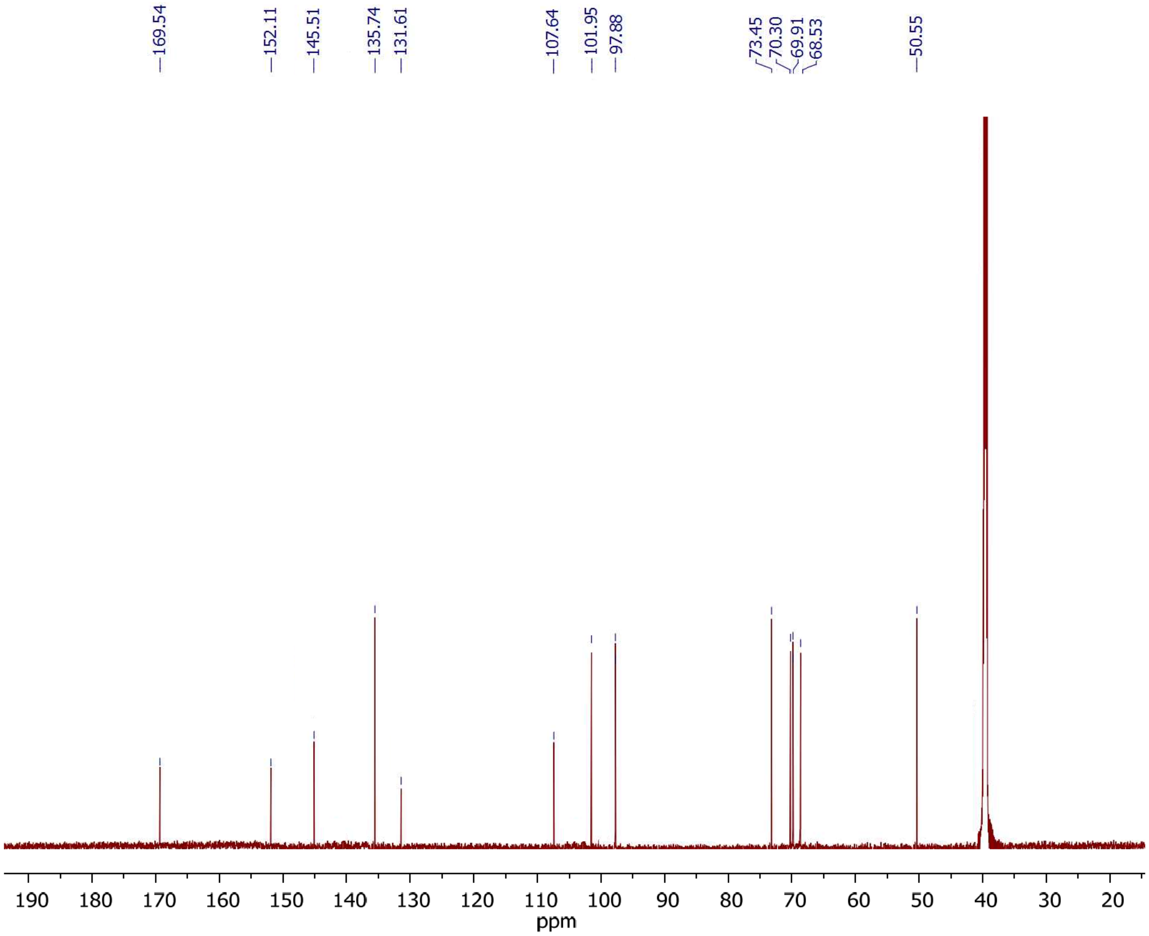

Figure S37. HPLC purification of Fraction C.

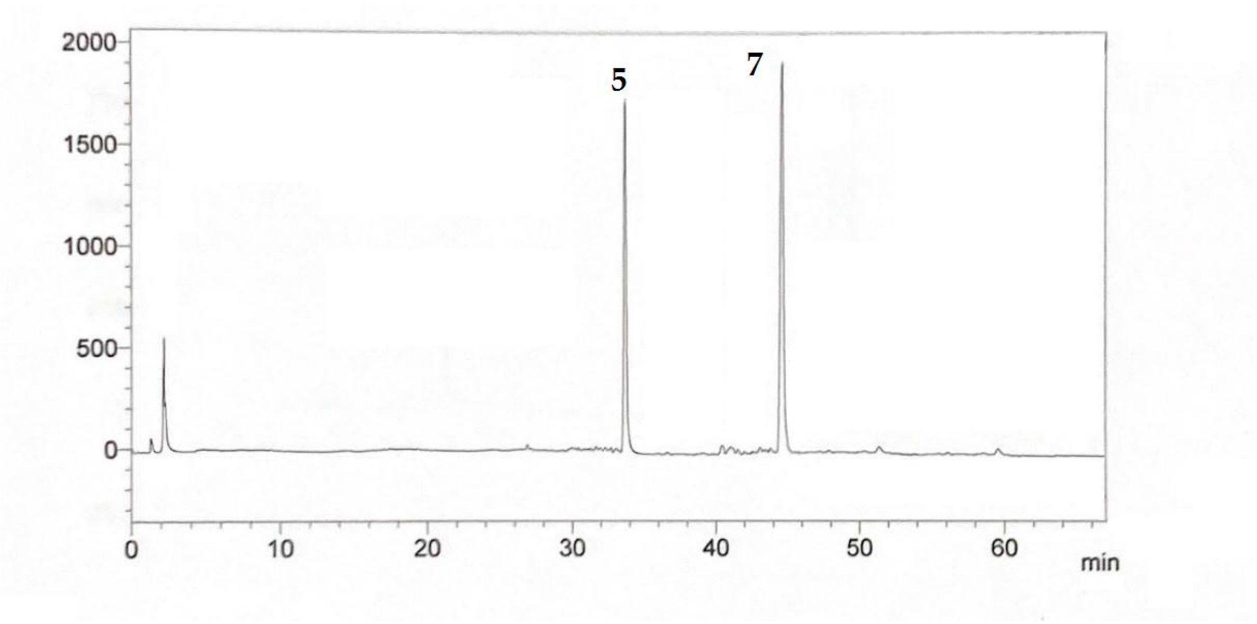

Figure S38. HPLC Purification of Fraction D.

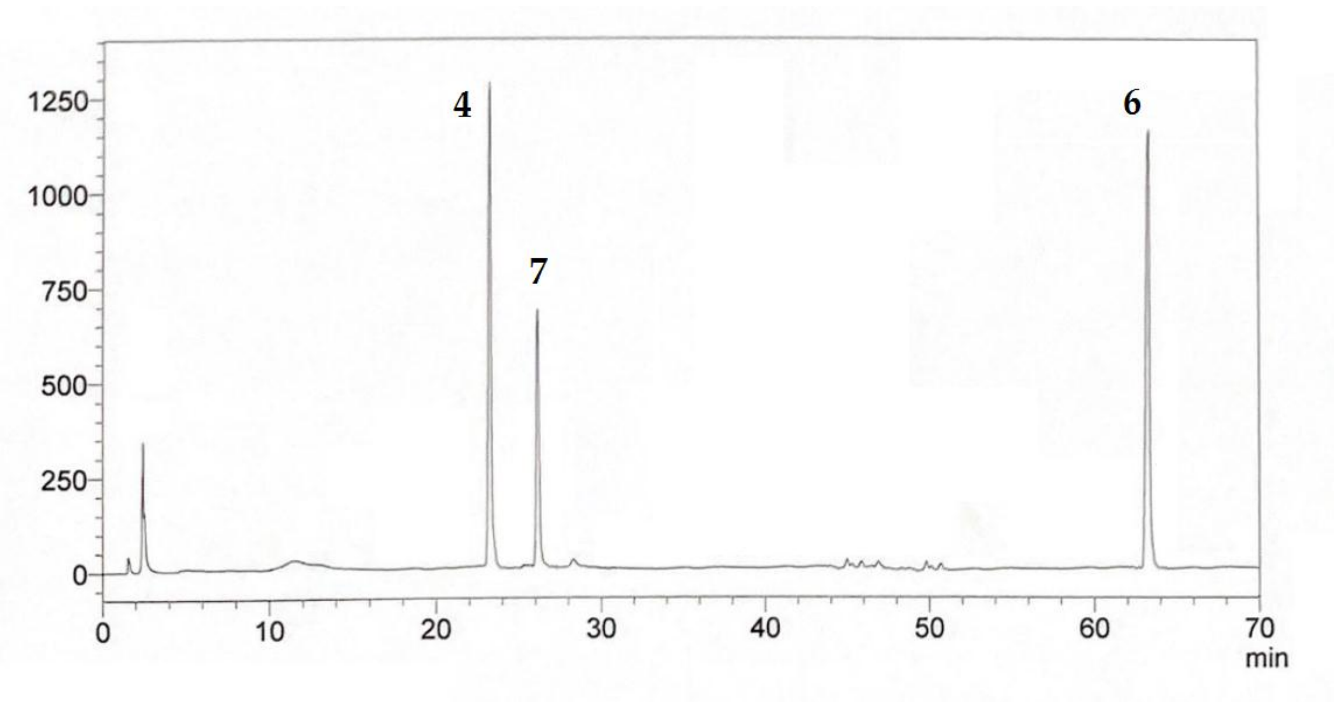

Figure S39. HPLC purification of Fraction E.

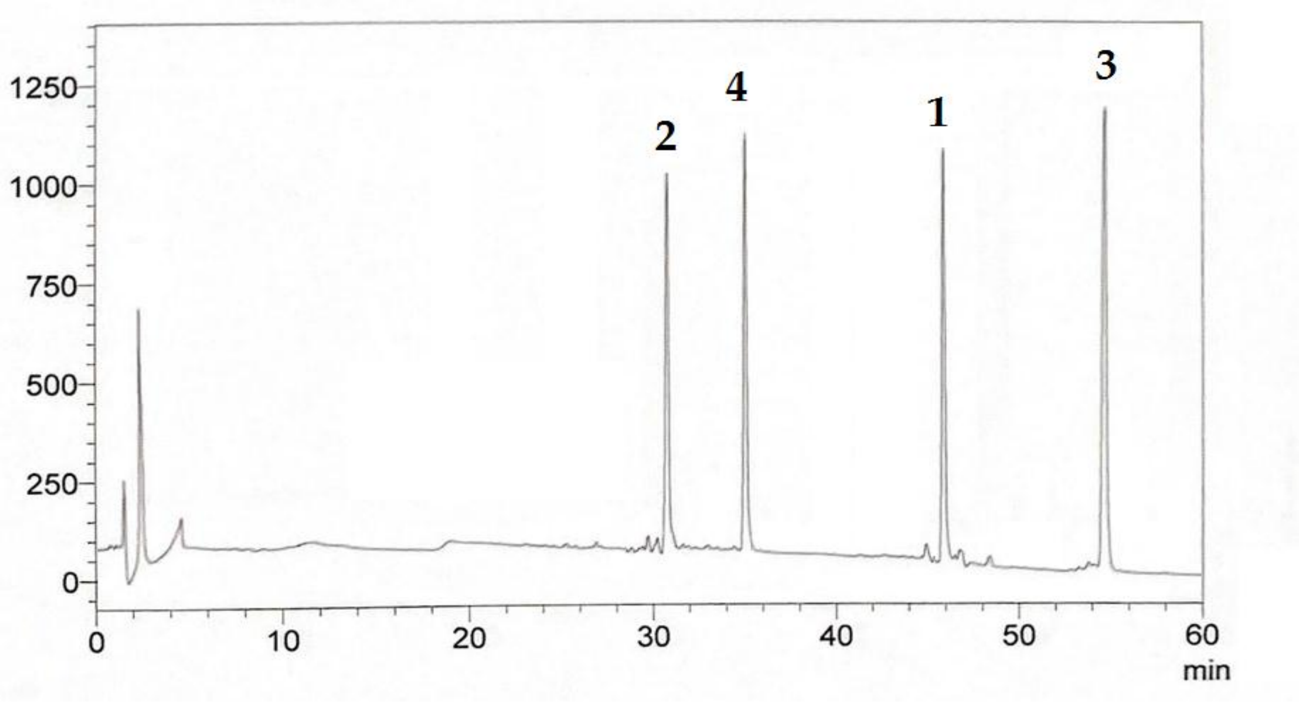

Supplement: Supplementary file 1 [file plants-11-00476-s001.zip › plants-1569312-supplementary.pdf]
